# The Bric-à-Brac BTB/POZ transcription factors are necessary in niche cells for germline stem cells establishment and homeostasis through control of BMP/DPP signaling in the *Drosophila melanogaster* ovary

**DOI:** 10.1371/journal.pgen.1009128

**Published:** 2020-11-05

**Authors:** Laurine Miscopein Saler, Virginie Hauser, Mathieu Bartoletti, Charlotte Mallart, Marianne Malartre, Laura Lebrun, Anne-Marie Pret, Laurent Théodore, Fabienne Chalvet, Sophie Netter

**Affiliations:** 1 Université Paris-Saclay, CEA, CNRS, Institute for Integrative Biology of the Cell (I2BC), Gif-sur-Yvette, France; 2 Université Paris-Saclay, UVSQ, CEA, CNRS, Institute for Integrative Biology of the Cell (I2BC), Gif-sur-Yvette, France; College de France CNRS, FRANCE

## Abstract

Many studies have focused on the mechanisms of stem cell maintenance *via* their interaction with a particular niche or microenvironment in adult tissues, but how formation of a functional niche is initiated, including how stem cells within a niche are established, is less well understood. Adult *Drosophila melanogaster* ovary Germline Stem Cell (GSC) niches are comprised of somatic cells forming a stack called a Terminal Filament (TF) and associated Cap and Escort Cells (CCs and ECs, respectively), which are in direct contact with GSCs. In the adult ovary, the transcription factor Engrailed is specifically expressed in niche cells where it directly controls expression of the *decapentaplegic* (*dpp*) gene encoding a member of the Bone Morphogenetic Protein (BMP) family of secreted signaling molecules, which are key factors for GSC maintenance. In larval ovaries, in response to BMP signaling from newly formed niches, adjacent primordial germ cells become GSCs. The *bric-à-brac* paralogs (*bab1* and *bab2*) encode BTB/POZ domain-containing transcription factors that are expressed in developing niches of larval ovaries. We show here that their functions are necessary specifically within precursor cells for TF formation during these stages. We also identify a new function for Bab1 and Bab2 within developing niches for GSC establishment in the larval ovary and for robust GSC maintenance in the adult. Moreover, we show that the presence of Bab proteins in niche cells is necessary for activation of transgenes reporting *dpp* expression as of larval stages in otherwise correctly specified Cap Cells, independently of Engrailed and its paralog Invected (En/Inv). Moreover, strong reduction of *engrailed/invected* expression during larval stages does not impair TF formation and only partially reduces GSC numbers. In the adult ovary, Bab proteins are also required for *dpp* reporter expression in CCs. Finally, when *bab2* was overexpressed at this stage in somatic cells outside of the niche, there were no detectable levels of ectopic En/Inv, but ectopic expression of a *dpp* transgene was found in these cells and BMP signaling activation was induced in adjacent germ cells, which produced GSC-like tumors. Together, these results indicate that Bab transcription factors are positive regulators of BMP signaling in niche cells for establishment and homeostasis of GSCs in the *Drosophila* ovary.

## Author summary

A stem cell is able to divide and produce two daughter cells, one of which retains stem cell status, the second, in contrast, becoming specialized for functions in the corresponding organ. Harnessing the potential of stem cells therefore represents opportunities for cell therapy upon organ damage. Maintenance of stem cells requires their interaction with specific cellular microenvironments called niches. Very little is known about how stem cell microenvironments are established during development and in pathological settings such as during cancer metastasis. The *Drosophila* adult ovary is probably the best characterized niche-stem cell system. It has been shown that niche cells secrete signaling proteins of the Bone Morphogenetic Protein (BMP) family, which by binding to receptors present at the membrane of adjacent Germline Stem Cells (GSCs), instruct these cells to maintain stem cell status. The analysis of ovaries mutant for the two *bric-à-brac* (*bab*) genes, showed that they are necessary within precursor cells for correct formation of GSC niches and for activation of the BMP pathway leading to the establishment of the first GSCs in the developing ovary. We have also found that Bab proteins contribute to GSC maintenance in the adult along with other transcription factors, Engrailed/Invected. Importantly, overproduction of one of the Bab proteins leads to the production of GSC tumors in the adult ovary underlining a role for this protein in GSC homeostasis.

## Introduction

A stem cell niche allows, first, the establishment of stem cells, and second, the maintenance of a balance between stem cell self-renewal and differentiation. Much more is known about stem cell maintenance than about initial stem cell establishment. The interactions between niche and stem cells need to be strictly controlled for maintaining homeostasis of adult tissues. In fact, a defect in stem cell homeostasis can be pathological in humans, producing for example cancer stem cells [1–3], and may also be an important part of premature aging when stem cell populations lose their potential to self-renew [4]. The discovery of pre-metastatic niches in cancer [5,6] also makes the study of the properties of stem cell niches a key for gaining headway in cancer biology.

**Fig 1:**
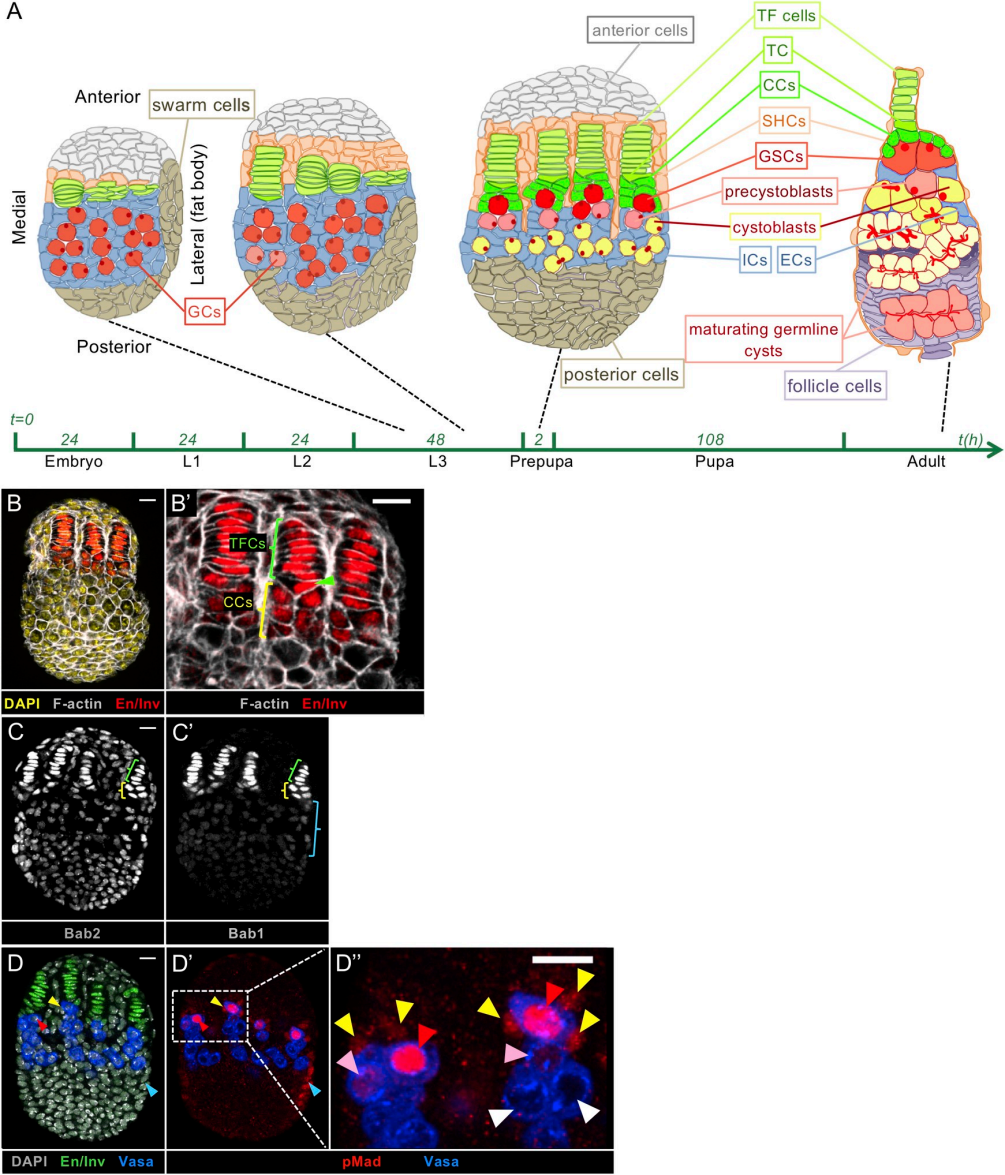
Specific cell types of the developing and adult *Drosophila* ovary. (A) Schematic drawings of a developing ovary from mid L3 to prepupal stages and of an adult germarium. Anterior is to the top and for the developing ovaries, medial is to the left. Intervals on the X-axis indicate the duration of each stage. The red, spherical structure in Germ Cells (GCs), Germline Stem Cells (GSCs) and precystoblasts represent the spectrosome and the red, branched structures in maturing germline cysts in the germarium represent fusomes, which are derived from spectrosomes. Spectrosomes and fusomes are cytoplasmic structures composed of cytoskeletal proteins. A fully-formed GSC niche is composed of a Terminal Filament (TF), which is a stack of about 8 flattened cells, approximately 5–6 Cap Cells (CCs) present at the base of the TF, one triangularly-shaped transition cell (TC) between the bottom of the TF and the CCs [10] and, in adult ovaries, the anterior Escort Cells (ECs) derived from larval posterior most-Intermingled Cells (ICs). At the prepupal stage, Sheath Cells (SHCs) begin to separate individual ovarioles having already begun with the TFs. (B-D”) Whole mount immunostaining of wild type prepupal ovaries. Anterior is up, medial is to the left. Scale bar: 10 μm. (B’,D”) Higher magnifications of the niche regions of the corresponding ovaries (B,D). (B,B') Engrailed/Invected (En/Inv) (red) mark the niche cell nuclei specifically and F-actin labeled with phalloidin (grey) marks all cell membranes. (B’) TF cells (green bracket) and the TC (green arrowhead) present nuclei accumulating high levels of En/Inv, while CCs (yellow bracket) present lower En/Inv levels. (C) Bab2 accumulates in the nuclei of all somatic cells, but at a higher level in TFs and CCs (green and yellow brackets, respectively). (C') Bab1 accumulates in TF cells and CCs, and is detected at low levels in ICs (blue bracket). (D) En/Inv (green) mark the nuclei of niche cells, and Vasa (blue) marks the cytoplasm of GCs. (D',D") In the most anterior GCs, the BMP signaling pathway is activated as evidenced by accumulation of pMad (red) at high (red arrowheads) or low (pink arrowheads) levels. GCs accumulating pMad and in contact with CCs are considered as GSCs. CCs (yellow arrowheads) and other peripheral posterior somatic cells (blue arrowhead) also accumulate pMad.

The *Drosophila melanogaster* adult ovary has proven to be an excellent model for understanding how interaction with adjacent somatic niche cells allows for maintenance of Germline Stem Cell (GSC) status [7,8]. Approximately 20 individual GSC niches, each associated with a small number of GSCs (2–3), are present in the *Drosophila* adult ovary at the tip of structures called germaria (Fig 1A). Each GSC niche is composed of several types of somatic cells: Terminal Filament (TF) cells, a triangularly-shaped transition cell (TC), Cap Cells (CCs) and the anterior Escort Cells (ECs) (Fig 1A) [9–11]. Both CCs and anterior ECs are in direct contact with GSCs. CCs are considered to be the key component of GSC niches, their number correlating closely with the number of GSCs [12,13]. CCs anchor GSCs to the niche by DE-cadherin-mediated adhesion [14] and produce two Bone Morphogenetic Proteins (BMPs), Decapentaplegic (Dpp) and Glass Bottom Boat (Gbb), acting as short-range secreted signals required for GSC maintenance [8,13,15–19]. Indeed, Dpp/Gbb signals are transduced in GSCs, which leads to the phosphorylation of the transcription factor Mad (pMad), its translocation into the nucleus and transcriptional repression of germline differentiation genes such as *bag-of-marbles* (*bam*) [18,20]. The homeobox transcription factor Engrailed (En), which is only expressed in niche cells in the ovary, binds *dpp cis*-regulatory sequences *in vitro* and activates *dpp* transcription in CCs allowing for GSC maintenance in the adult ovary [17]. Several other signaling pathways, such as the JAK/STAT [19,21] and Hedgehog [16,22,23] pathways, are active in different niche cells types and have also been implicated in regulation of *dpp* expression in these cells. In addition, ectopic expression of *dpp* or *engrailed* and ectopic activation of JAK/STAT signaling in non-niche somatic cells of the germarium, lead to a germarial GSC-like tumorous phenotype further supporting the implication of these factors in GSC homeostasis [8,18,19,24,25].

Morphogenesis of GSC niches occurs in the third instar larval ovary beginning with TF formation which involves flattening, sorting, intercalation and stacking of somatic TF cell precursors, initiating at the medial side of the ovary and progressing as a wave laterally (Fig 1A) [26,27]. Individualization of each TF is accomplished by the migration of apical somatic cells between TFs (Sheath Cells, Fig 1A) [28]. The number of TFs that form in the larval ovary (18–20) corresponds to the number of GSC niches at the adult stage [29–31]. At the prepupal stage, at the base of each newly formed TF, the anterior-most Intermingled Cells (ICs) differentiate into CCs adopting a cuboidal shape clearly distinguishable from that of TF cells (Fig 1A–1B') [10,32]. At this stage, the transition cell is already distinguishable (Fig 1A–1B') [10]. Posterior-most ICs will give rise to ECs in adult ovaries (Fig 1A) [33].

Only a few genes have been reported to be implicated in TF formation, notably, the *bric-à-brac* paralogs (*bab1/bab2*) [26,27,29,34,35] and the *engrailed*/*invected* paralogs (*en/inv*) [36,37] (Fig 1B–1D). The *bab1* and *bab2* genes encode proteins sharing evolutionarily conserved domains: a BTB (Broad-Complex, Tramtrack and Bric-à-brac)/POZ (POx virus and Zinc finger) domain involved in homodimeric and heterodimeric interactions [38] and a Bab-Conserved Domain (BabCD) involved in Protein-DNA interactions [34,39]. In the larval ovary, Bab1 has been reported to be present only in niche cells and Bab2 in all somatic cells, however at higher levels in niche cells [34] (Fig 1C and 1C’). Heterozygosity for strong or null alleles of both *bab* genes leads to a dominant phenotype characterized by an excess of TFs, resulting in an excess of GSC niches in adults [29], and by a recessive phenotype characterized by a defect in TF formation associated with production of atrophied ovaries with few germ cells in adults and sterility [26,27]. The *en/inv* paralogs are only expressed in GSC niche cells in larval and adult ovaries [31,40–42]. Induction of TF cell clones homozygous for a deletion encompassing both paralogs identified a function for these genes within TF cells for their correct alignment to form straight TFs [36]. CC specification has been shown to require the combined action of Notch signaling and the large Maf transcription factor Traffic Jam (Tj) [10,12,40,43,44].

The newly formed GSC niches become functional as of the prepupal stage [32]. Before niche formation, all Primordial germ Cells (PGCs) have been shown to exhibit BMP signaling activation [45,46], which is correlated with detection of Dpp in all somatic cells of the ovary [47]. During larval stages, Dpp signaling prevents PGC differentiation into germline cysts [32,45,48] and promotes PGC proliferation [32,47]. Upon GSC niche formation starting at the late third instar larval stage, *dpp* expression [32,48] and Dpp protein accumulation [47], become unevenly distributed in the ovary, with highest levels found in niche cells. Among PGCs, only those in contact with the Dpp-secreting niche cells retain BMP signaling activation as evidenced by the presence of pMad (Fig 1D–1D") [40] and become functional GSCs [32]. The more posterior PGCs, not in contact with niche cells, lose Dpp signaling activation (Fig 1D") and proceed to differentiate into cystoblasts, whereupon they produce the first germline cysts [32,48,49].

In this study, we addressed the roles of *bab1* and *bab2* specifically within niche cells for several aspects of functional GSC niche formation. Indeed, it has previously been proposed that the small size of adult germaria in *bab* mutants in which Bab proteins are depleted from all somatic ovarian cells might indicate a decrease in the number of GSCs produced [27]. We used a strong hypomorphic allele of *bab1* (*bab*^*A128*^) and Gal4-targeted RNA interference (RNAi) for efficient knockdown of each or both of the two paralogs specifically in GSC niches during their formation in larvae. This approach allowed us to demonstrate that drastic reduction of both Bab1 and Bab2 levels only within precursor niche cells inhibits TF formation. Surprisingly, cells exhibiting several CC characteristics were nonetheless present upon depletion of Bab proteins in developing niches. In addition, we have identified a new essential role for *bab* genes in prepupal niches for initial establishment of GSCs correlated with a role in ensuring *dpp* expression in CCs, as assessed by two *dpp* reporter transgenes. This function of *bab* in CCs is unlikely to require that of the *en*/*inv* genes since their expression levels were not affected when Bab1 and Bab2 were depleted. We also have evidence suggesting that En/Inv may not be essential for activation of BMP signaling and GSC establishment in the larval ovary, contrasting with their known essential functions in adult ovaries for BMP-mediated GSC maintenance. In adult ovaries, we show that Bab proteins contribute to GSC maintenance and activate a transgene reporting *dpp* expression in CCs. Finally, when *bab2* was overexpressed outside of niche cells in somatic ovarian cells at the adult stage, germaria with a large excess of GSC-like cells forming tumors were produced. This was associated with ectopic somatic activation of a transgene reporting *dpp* expression and BMP signaling activation in GCs without ectopic expression of *en/inv* in the *bab2*-overexpressing somatic ovarian cells. Together, our results indicate that *bab* gene functions play a major role in niche cells for GSC establishment in the larval ovary and contribute to GSC maintenance in the adult, both likely via positive regulation of *dpp* expression in CCs.

## Results

### Reduction of Bab1 and Bab2 specifically in GSC niche cells during larval stages leads to abnormal TF formation

**Fig 2:**
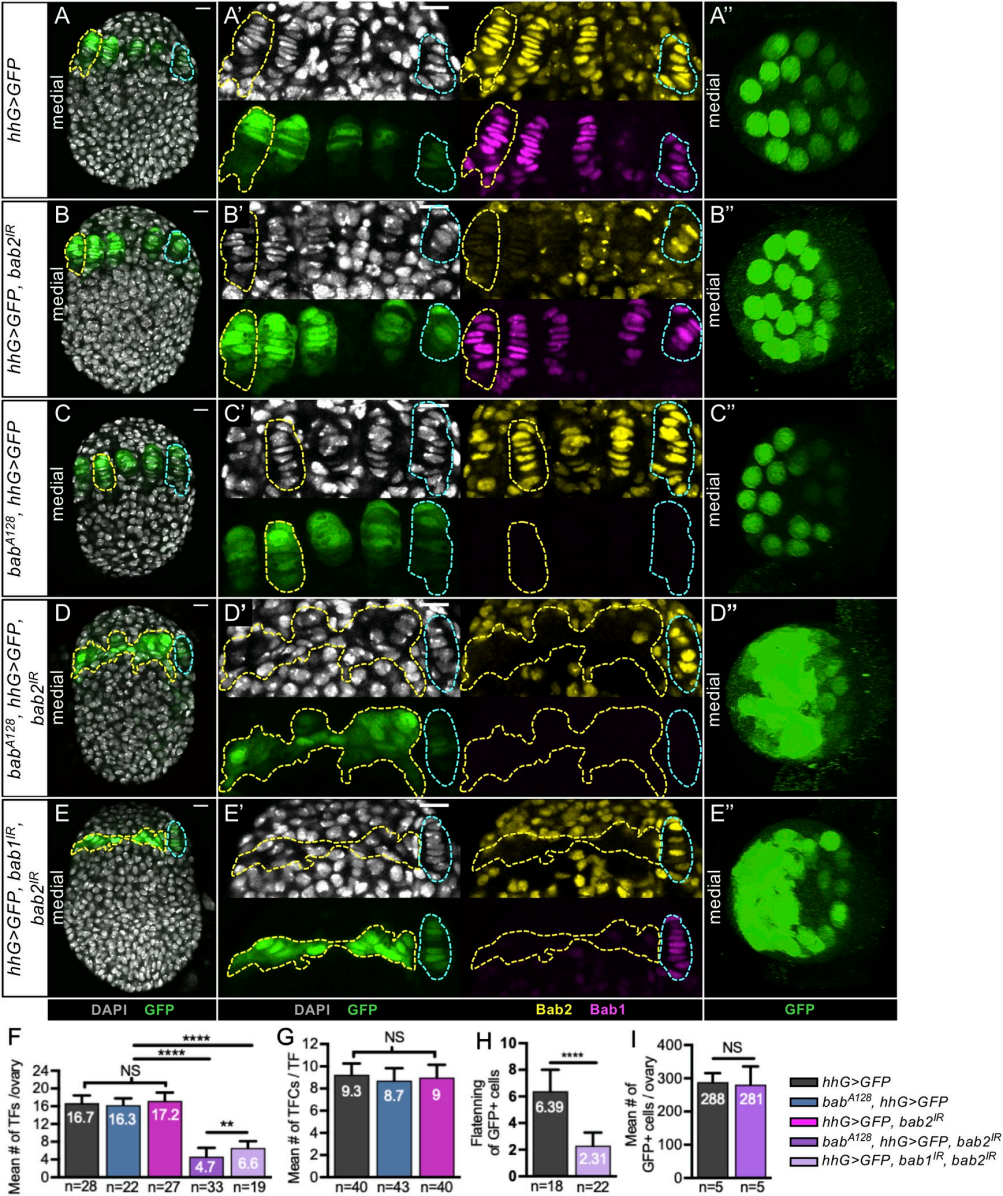
Reduction of both Bab1 and Bab2, but not each separately, in developing niches impedes Terminal Filament formation in prepupal ovaries. (A-E) Whole mount prepupal ovaries immunostained for detection of GFP (green), Bab1 (magenta) and Bab2 (yellow). Nuclei are labeled with DAPI (grey). The yellow dotted lines encircle GSC niches in the medial region of the ovary, and the blue dotted lines indicate laterally positioned niches. Anterior is up, medial is left. Scale bars: 10 μm. (A’,B’,C’,D’,E’) Higher magnifications of the niche regions of the corresponding ovaries. (A”,B”,C”,D”,E”) Anterior views of ovaries using 3D reconstruction. Each GFP circle corresponds to the cross section of one TF. (A-A’) Control ovary expressing a *UAS-GFP* construct under the control of a *hedgehog(hh)-Gal4 driver (hhG>GFP)*. Bab2 accumulates in nuclei of all somatic ovarian cells, but at a higher level in TF cell and Cap Cell (CC) nuclei. Bab1 accumulates at a high level in TF cell and CC nuclei. (B-B’) Prepupal ovary expressing an RNAi transgene against *bab2 (hhG>GFP*, *bab2*^*IR*^*)*. Consistent with the expression profile of the *hhG* driver, Bab2 depletion is obtained in all medial TF cells but not in all CCs at the base of these TFs, and is not obtained in lateral *hhG+* cells. (C-C’) Prepupal ovary from a female homozygous for a *bab1* mutant allele (*bab*^*A128*^, *hhG>GFP)*. Bab1 is not detectable in medial or lateral niches, or in underlying ICs. (D-D’) Prepupal ovary homozygous for *bab*^*A128*^ and expressing an RNAi transgene against *bab2* (*bab*^*A128*^, *hhG>GFP*, *bab2*^*IR*^*)*. Bab2 depletion is obtained in medial but not lateral *hhG+* cells. Bab1 is not detectable. (E-E’) Prepupal ovary expressing RNAi transgenes against *bab1* and *bab2* (*hhG>GFP*, *bab1*^*IR*^, *bab2*^*IR*^*)*. Bab1 and Bab2 depletion is obtained in medial but not lateral *hhG+* cells. (D-D’,E-E’) Upon reduction of both Bab1 and Bab2 in *hhG+* cells (yellow dotted lines), these cells fail to flatten, to stack and to form TFs. (F-I) Graphs comparing different parameters related to TF formation. (F) The mean number of TFs per ovary is not significantly different between the control and the ovaries depleted of either Bab1 or Bab2. However, the ovaries depleted of both Bab1 and Bab2 have significantly fewer TFs per ovary than the control. The higher penetrance evidenced by the abnormally low TF number per ovary phenotype using *bab*^*A128*^ instead of *UAS-bab1*^*IR*^ for the double *bab1/bab2* knockdown may be attributable to the more efficient depletion obtained for Bab1 with *bab*^*A128*^ than with *UAS-bab1*^*IR*^. (G) The mean number of TF cells (TFCs) per TF is not different between control ovaries and ovaries depleted of Bab1 or Bab2. (H) Control *hhG+* cells are significantly flatter than those knocked down for *bab1* and *bab2*, with the degree of flattening measured as the ratio between the width and height of the cells using F-actin labeling to visualize cell perimeters. (I) The mean number of *hhG+* cells per ovary is comparable in control ovaries and ovaries depleted of Bab1 and Bab2. Values are presented as means +s.d., p-values are calculated using a one-way ANOVA test for F and G, and a two-tailed t-test for H and I. n: sample size; NS: Not Significant (p>0.05); **** (p<0.0001).

Although the two *bab* genes have been shown to be necessary for TF formation [26,27], we aimed at addressing their roles specifically within niche cells for this process. We thus tested the effect of depletion of each of the Bab proteins individually or together in TFs and CCs during niche formation. As a control, we used the *hedgehog-Gal4* (*hhG*) driver combined with a *UAS-GFP* reporter (*hhG>GFP*) in order to indicate the cells in which the driver is active. We found specific expression of GFP in a subset of anterior somatic cells close to PGCs in L2 stage ovaries (S1A Fig) through to late L3 ovaries whereupon GFP was expressed in fully-formed TFs [33,50] (S1B–S1D Fig). In these prepupal ovaries, much higher GFP accumulation in medial vs. lateral niches was noted (Fig 2A–2A”’). Inside each niche, *hhG>GFP* expression levels were mosaic between TF cells and always low in CCs (Fig 2A and 2A').

In these control *hhG* ovaries, Bab2 was detected in all somatic cells at L2 and L3 stages (S1E–S1H Fig), and at higher levels in TFs and CCs in prepupae (Fig 1C and [34]). When the *hhG* driver was used to express a *bab2*-specific RNAi construct (*hhG>bab2*^*IR*^), efficient Bab2 depletion was observed in most, but not all, *hhG+* cells of L2 and midL3 ovaries (S1I–S1I" and S1J–S1J" Fig). By the prepupal stage, Bab2 depletion was observed in all medial TF cells, but not in all CCs, nor in lateral *hhG+* cells consistent with low *hhG* expression in CCs and lateral TFs (Fig 2B', and S4B and S4B’ Fig). In this context, the formation of TFs is not affected (Fig 2B, 2B’, 2F and 2G). In contrast, *bab2* knockdown in all ovarian somatic cells during niche formation, using the *bab-Gal4* driver (hereafter named *babG*, S2 Fig), led to severe overall ovarian morphogenetic and growth defects (S3A Fig and [51]) indicating an essential role for *bab2* in non-niche cells for ovarian development and rendering it impossible to study niche formation in this context.

Bab1 accumulation in control *hhG>GFP* ovaries was only detected as of mid L3 ovaries in cells beginning to form TFs (S1A–S1D’ Fig). By the prepupal stage Bab1 was detected at high levels in both TF cells and CCs (Fig 1C') as previously reported [34]. In addition, we report that low levels of Bab1 are also detected in ICs at this stage (Fig 1D'). For *bab1* depletion, we used *bab*^*A128*^ reported as a *bab1* strong hypomorphic allele [34,52]. Females homozygous for *bab*^*A128*^ had undetectable levels of Bab1 in prepupal ovaries (Fig 2C'). Like for Bab2, Bab1 depletion did not affect several parameters for normal TF formation (Fig 2C, 2C’, 2F and 2G). Importantly, we did not detect any cross-regulation between *bab* genes at the prepupal stage (Fig 2A’–2C’). From these results, we conclude that efficient depletion of either Bab1 or Bab2 in niche cells throughout ovary development does not affect niche morphogenesis.

Next, to test the effect of knocking down *bab1* and *bab2* at the same time in niche cells, we used four genetic contexts: (1) *bab*^*A128*^,*hhG>GFP*,*bab2*^*IR*^, (2) *hhG>GFP*,*bab1*^*IR*^,*bab2*^*IR*^, (3) *bab*^*A128*^,*hhG>GFP*,*bab1-bab2*^*shmiR*^ (chained shmiR allow to co-express *bab1* and *bab2* shmiRs, [53]) and (4) *hhG>GFP*,*bab1-bab2*^*shmiR*^. In prepupal ovaries from females of the first three genotypes, the medial part of the prepupal ovary contained a large cluster of *hhG+* cells strongly depleted of both Bab1 and Bab2, which failed to form TFs (Fig 2D–2E' and S4C and S4C’ Fig). The fourth genotype, *hhG>GFP*,*bab1-bab2*^*shmiR*^ did not produce a clear mutant prepupal ovary phenotype likely due to inefficient extinction of *bab1* (S4B and S4B’ Fig). The defects observed on niche morphogenesis upon depletion of both Bab proteins during larval stages are consistent with the previously-described phenotypes of *bab* gene mutants [26,27,34]. We quantified the number of TFs per ovary (Fig 2F), the number of cells per TF (Fig 2G) and the extent of flattening (ratio of the width to height of the cell) of the medial *hhG+* cells since flattening is a hallmark of TF cell differentiation (Figs 1B’ and 2H) and found that these parameters were significantly lower than those in the control, while the overall number of *hhG+* cells per ovary was not affected (Fig 2I). In the lateral-most part of the ovary, where *hhG* driver expression was low, low depletion of Bab proteins was consequently obtained and *hhG+* cells formed apparently normal TFs (Fig 2D, 2D’, 2E and 2E’).

Therefore, efficient reduction of both Bab1 and Bab2 specifically in niche cells, using different genetic tools, impaired TF formation significantly, while reduction of each one individually did not. Our results also indicate that reduction of both Bab proteins did not affect the overall number of precursor niche cells. Together, these results indicate that Bab proteins are necessary specifically in these cells for the morphogenetic processes involved in TF formation during larval stages.

### Reduction of Bab1 and Bab2 levels specifically in GSC niche cells during larval stages leads to loss of GSC establishment

**Fig 3:**
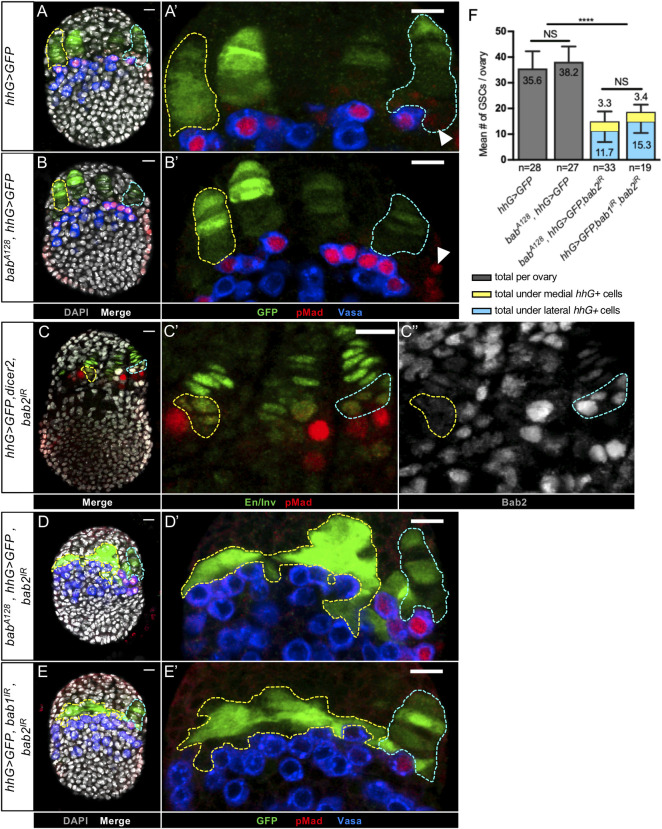
Reduction of both Bab1 and Bab2, but not each separately, in developing niches of larval ovaries leads to almost no Germline Stem Cell establishment by the prepupal stage. (A-E) Prepupal ovaries immunostained for GFP to mark niche cells (green), Vasa (blue) to mark germ cells (GCs) and pMad (red) to mark GSCs. Nuclei are labeled with DAPI (grey). Anterior is up, medial is left. Scale bars: 10 μm. The yellow dotted lines encircle niches (A-C) or *hhG+* cells depleted of Bab1 and Bab2 (D-E) of the medial region of the ovaries while the blue dotted lines encircle niches of the lateral region. (A’,B’,C’,C”,D’,E’) Higher magnifications of the niche regions of the corresponding ovaries. (A-A’) In a control ovary, pMad is detected in the anterior-most Germ Cells (GCs). These Vasa+/pMad+ GCs are in direct contact with the niches and are considered as Germline Stem Cells (GSCs). (B-B') In an ovary mutant for *bab1* (*bab*^*A128*^, *hhG>GFP*), no significant difference is observed when compared to the control regarding the presence of GSCs in contact with the niches. (A’,B’) Cap Cells (CCs) and some peripheral posterior somatic cells (arrowheads) accumulate pMad, but can be distinguished from GCs since they do not accumulate Vasa. (C-C”) In a *hhG>GFP*,*dicer2*,*bab2*^*IR*^ ovary, the rare medial niches strongly depleted of Bab2 in all CCs (yellow dotted lines) are associated with GSCs, as are lateral niches not depleted of Bab2 (blue dotted lines). (D-E’) In *bab*^*A128*^,*hhG>GFP*,*bab2*^*IR*^ and *hhG>GFP*, *bab1*^*IR*^,*bab2*^*IR*^ prepupal ovaries, almost none of the GCs in contact with *hhG+* medial cells, strongly depleted of Bab1 and Bab2, are GSCs since they are not pMad+. In contrast, in the same ovaries, in lateral niches where Bab depletion does not occur, normal niches are formed and are associated with GSCs as in the control. (F) Graph comparing the mean number of GSCs per ovary in control ovaries (*hhG>GFP*) and in ovaries strongly depleted of Bab1 alone (*bab*^*A128*^,*hhG>GFP*) or both Bab1 and Bab2 (*bab*^*A128*^,*hhG>GFP*,*bab2*^*IR*^ and *hhG>GFP*, *bab1*^*IR*^,*bab2*^*IR*^). A significantly lower number of GSCs per ovary was obtained only when Bab1 and Bab2 were depleted together. Moreover, in both mutant contexts, the majority of GSCs were found in the lateral part of the ovary were TFs form (blue bar) and very few in the medial part where they do not form (about 3 GSCs per ovary, yellow bar). Values are presented as means +s.d., p-values are calculated using a one-way ANOVA test. n: sample size; NS: Not Significant (p>0.05); **** (p<0.0001).

We used the same genetic approaches as those described in the previous section to deplete Bab1 and/or Bab2 proteins from niche cells, and then asked whether the resulting disorganized *hhG+* cells were able to recruit the initial GSCs during the larval to prepupal transition. To test for functional niche activity of *hhG+* cells, BMP pathway activation in adjacent GCs was used as a characteristic of GSC identity. For this, accumulation of a direct downstream component of BMP pathway activation, phosphorylated Mad (pMad), was monitored in GCs. In control prepupal ovaries, pMad was detected in anterior-most GCs adjacent to niches at either high or low levels (Figs 1D', 1D", 3A and 3A' and S4D and S4D’ Fig). In the *bab*^*A128*^ mutant context, pMad+ GSCs were present at the base of TFs (Fig 3B and 3B') and at the same mean number per ovary as that in the control (Fig 3F). In *hhG>GFP*,*dicer2*,*bab2*^*IR*^ ovaries, only very rare niches in which all CCs presented undetectable levels of Bab2 were obtained (Fig 3C"), and in these cases, adjacent GCs were always positive for pMad just as in Bab2+ niches (Fig 3A, 3A’, 3C and 3C’). These results indicate that strong reduction of either Bab1 or Bab2 individually in niche cells did not affect GSC establishment.

In prepupal ovaries, upon efficient reduction of both Bab1 and Bab2 in *hhG+* cells, GCs were found in close contact with medial *hhG+* cells, but almost all of these GCs were devoid of pMad staining (Fig 3D–3E', and S4D–S4E’ Fig). In contrast, in the lateral region where normal TFs were formed, pMad+ GCs (*i*.*e*. GSCs) were found at the base of each niche, as in control ovaries (Fig 3A, 3A’ and 3D–3E', and S4D–S4E’ Fig). In the Bab1 and Bab2 depleted ovaries, the mean number of GSCs per ovary was significantly lower than in the control and, when present, these GSCs were associated with lateral TFs in almost all cases (Fig 3F). Thus, when Bab1 and Bab2 levels were efficiently reduced in *hhG+* niche cells using three different genetic contexts, GCs were present next to *hhG+* cells, but the BMP pathway was, for the most part, not activated in these cells, indicating that GSC status was compromised.

Close contact between CCs and GSCs via E-Cadherin (E-Cadh) junctions has been shown to be important for GSC maintenance, and E-Cadh accumulation between CCs and GSCs was shown to begin at the late L3 stage [18]. We thus analyzed the levels of E-Cadh between *hhG+* cells and adjacent GCs in *hhG>GFP*,*bab1*^*IR*^,*bab2*^*IR*^ prepupal ovaries. In the normally-formed lateral niches, E-Cadh was observed between niche cells and underlying GCs, as is also the case in the medial niche region, even though properly structured niches were not present (S5 Fig). Together, our results indicate that *bab1* and *bab2* functions are necessary in the developing niche for GSC establishment by the prepupal stage and suggest that these functions are not likely related to deregulation of E-cadherin-mediated adherens junction contact between niche cells and GSCs.

**Fig 4:**
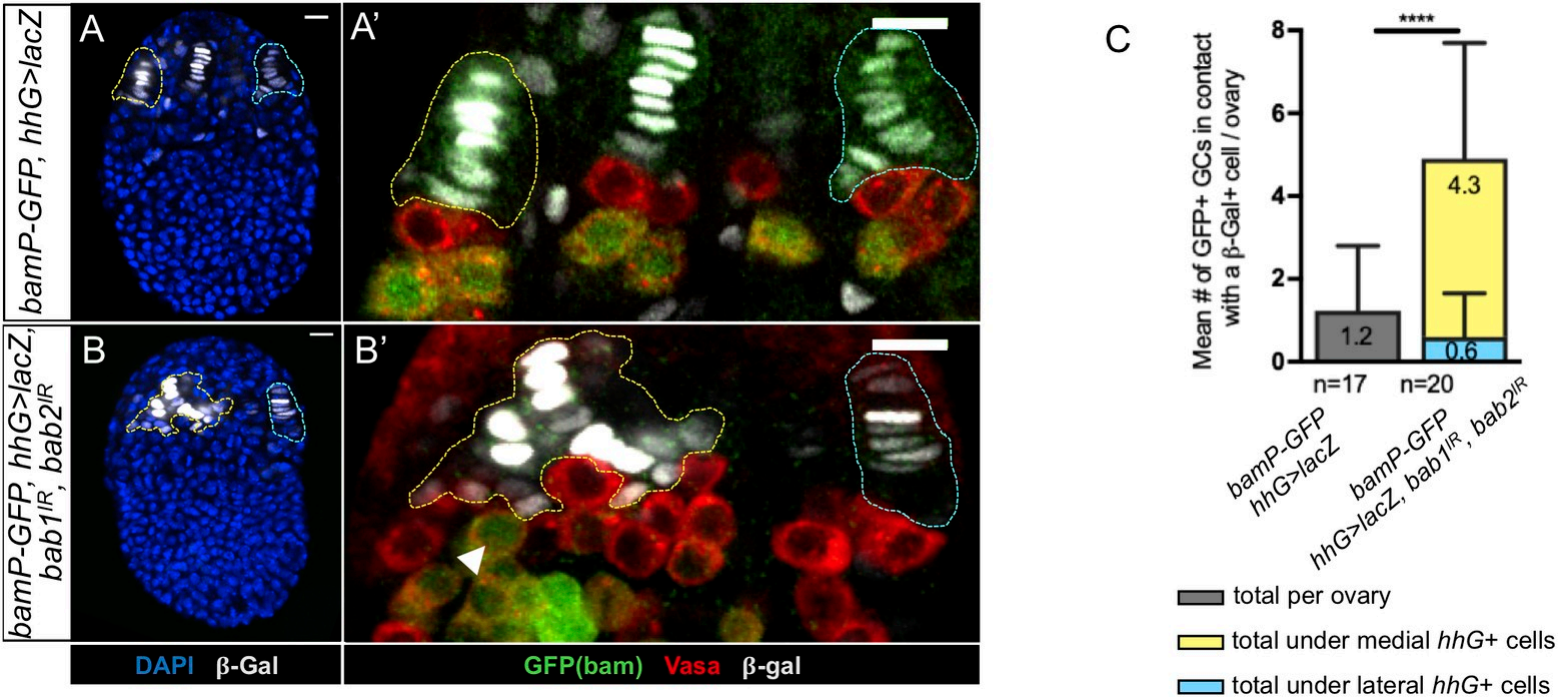
Some Germ Cells adjacent to niches with undetectable levels of Bab proteins begin to differentiate into cystoblasts. (A-B’) Prepupal ovaries immunostained to reveal β-Galactosidase (β-Gal, grey) for *hhG+* cells, GFP (green) for *bamP-GFP* transcriptional reporter expression, and Vasa (red) for Germ Cells (GCs). Nuclei are labeled with DAPI (blue). Anterior is up, medial is left. Scale bars: 10 μm. *hhG*+ cells are encircled in yellow and blue dotted lines in the medial and lateral regions of the ovaries, respectively. (A’,B’) Higher magnifications of the niche regions of the corresponding ovaries in (A,B). (A') In a control ovary, the differentiating GCs expressing the *bamP-GFP* transcriptional reporter are mainly found one-cell diameter away from the β-Gal+ niche cells. A faint GFP signal is also sometimes observed in some GSC niche cells. (B') In ovaries depleted of Bab1 and Bab2, some differentiating GCs can be observed in direct contact with β-Gal+ cells (white arrowhead). (C) Quantification of the mean number of GFP+ differentiating GCs in contact with β-Gal+ cells per ovary. In the ovaries depleted of Bab1 and Bab2, significantly more GFP+ differentiating GCs are found in contact with β-Gal+ cells and most of these cells are found in the medial part of the ovary where depletion of the Bab proteins is efficient (yellow bar), rather than laterally where it is not (blue bar). Values are presented as means +s.d., p-values are calculated using a t-test with Welch’s correction. n: sample size; **** (p<0.0001).

We next asked whether GCs next to Bab1- and Bab2-depleted niche cells devoid of pMad are driven to differentiate precociously. Germline cell differentiation was monitored by the expression of a *GFP* transcriptional reporter for *bam* expression (*bamP-GFP*) [54]. In control prepupal ovaries (*bamP-GFP*, *hhG>lacZ*), GCs that differentiate (GFP+/Vasa+) were almost never detected among GCs contacting *hhG+* (β-Gal+) niche cells (Fig 4A, 4A' and 4C), whereas all GCs located one-cell diameter away from these niche cells expressed this differentiation marker (Fig 4A'). In contrast, differentiating GCs were found significantly more frequently in direct contact with clusters of disorganized *hhG+* (β-Gal+) cells with undetectable Bab1 and Bab2 in the medial zone of prepupal ovaries (Fig 4B'). Abnormal differentiation of some GCs adjacent to the cluster of medial *hhG+* Bab1 and Bab2 depleted cells thus correlated with the absence of the GSC marker pMad in the same region. Taken together these results indicate that the functions of *bab1* and *bab2* are necessary in niches for acquisition of GSC status by adjacent PGCs as marked by sustained activation of the BMP pathway and absence of expression of the differentiation reporter *bamP-GFP*.

### Reduction of Bab1 and Bab2 levels specifically in GSC niche cells during larval stages does not alter expression of several Cap Cell markers at the prepupal stage

We next tested whether Bab proteins are necessary for specification of CCs, which are known to be essential for GSC establishment and maintenance [10,12,13,55]. We characterized the nature of the *hhG+* cells upon efficient reduction of Bab1 and Bab2 in these cells using a combination of markers that allow the distinction to be made between TF cells and CCs in prepupal niches: Traffic Jam (Tj) [56], *P1444-LacZ* [10], Delta [10,12] and *E(spl)mβ-CD2* [57].

**Fig 5:**
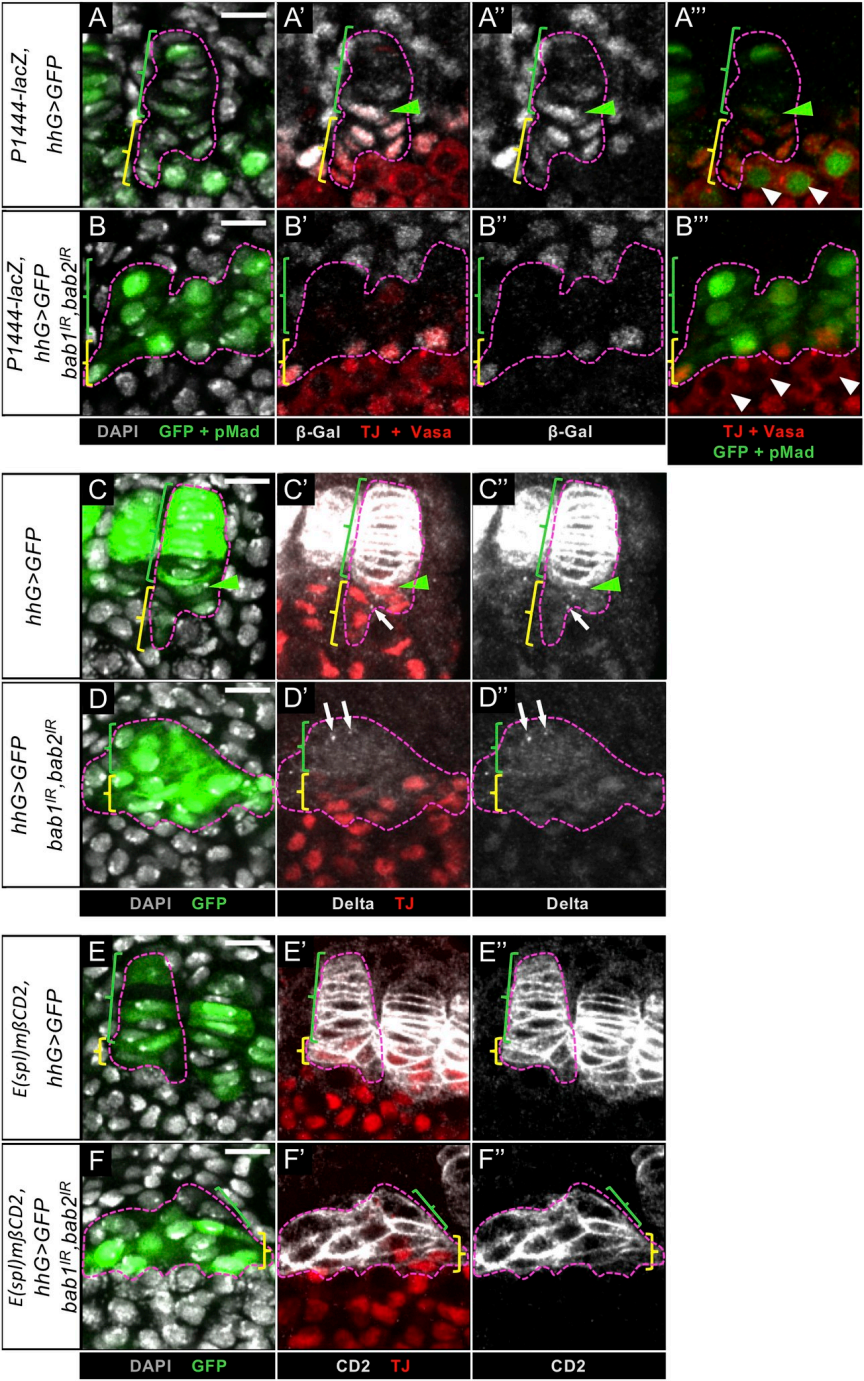
*bab1* and *bab2* functions are not necessary in Cap Cells for expression of several specific markers at the prepupal stage. (A-F”) Medial region of prepupal ovaries. Nuclei are labeled with DAPI (grey). Anterior is up. Scale bars: 10 μm. (A-A”’,C-C”,E-E”) *hhG>GFP* control ovaries. One niche is encircled in each panel (pink dotted line). The green and yellow brackets indicate Terminal Filament (TF) cells and Cap Cells (CCs), respectively, and the green arrowheads point to transition cells. (B-B”’,D-D”,F-F”) *hhG>GFP*,*bab1*^*IR*^,*bab2*^*IR*^ knockdown ovaries. The entire cluster of *hhG+* cells strongly depleted of Bab1 and Bab2 is encircled in each panel (pink dotted line). The green and yellow brackets indicate the anterior- and posterior-most *hhG+* cells, respectively. (A-A”’) In a control ovary, the two nuclear CC markers, *P1444-lacZ* (grey) and Traffic Jam (Tj, red), are detected in CCs and sometimes in the transition cell. *P1444-lacZ* is also detected at a low level in some TF cells. The Germ Cells (GCs) marked with Vasa (red) in direct contact with niche cells (white arrowheads) and showing nuclear pMad (green) are considered as Germline Stem Cells (GSCs). (B-B”’) Posterior-most medial *hhG+* cells depleted of Bab1 and Bab2, express both Tj and *P1444-lacZ* and are adjacent to GCs, which do not present pMad+ (B”’, arrowheads). (C-C") In a control ovary, Delta (grey) accumulating at the plasma membrane and in cytoplasmic vesicles in TF cells is also detected in vesicles around the transition cell (green arrowhead) and sometimes in CCs (arrow). (D-D") Upon depletion of Bab1 and Bab2 in *hhG+* cells, Delta is not present at the plasma membrane, but is found in some vesicles (arrows). (E-E",F-F") In both the control (E-E") and upon depletion of Bab1 and Bab2 in *hhG+* cells (F-F"), the Notch pathway transcriptional reporter *E(spl)mβ-CD2* is expressed since CD2 (grey) accumulates at the plasma membrane of TF cells and anterior *hhG+* cells, respectively, as well as in CCs and posterior *hhG+* cells, respectively, the latter also accumulating Tj.

Within normal niches, high levels of Tj protein and *P1444-lacZ* expression designate CCs, while low levels of *P1444-lacZ* expression without any Tj distinguishes TF cells [10](Fig 5A–5A”’ and 5C’). Upon efficient reduction of Bab1 and Bab2 by RNAi specifically in niche cell precursors during larval stages, the disorganized clustered medial *hhG+* cells were composed of two cell populations in prepupal ovaries. One of the populations was posteriorly-positioned, adjacent to GCs, and presented Tj nuclear accumulation (Fig 5B’–5B”’ and 5D’), associated with the expression of *P1444-lacZ* (Fig 5B’ and 5B”), like control CCs, except that the latter expressed higher levels of *P1444-lacZ*. Therefore, in posterior *hhG+* cells, these CC markers were present even though Bab proteins were reduced to undetectable levels. The second population of cells within the medial *hhG+* cell cluster, which was positioned anteriorly, did not accumulate Tj, like control TF cells (Fig 5B’ and 5D’), but unlike control TF cells, did not express even low levels of *P1444-lacZ* (Fig 5B’ and 5B”). The identity of this second population of cells is thus not that of normal TF cells.

We next tested for the presence of Notch pathway-associated niche markers, *i*.*e*. the Notch ligand, Delta (Fig 5C–5D”), and a Notch transcriptional reporter, *E(spl)mβ-CD2* (Fig 5E–5F”). In *hhG+* cells knocked down for *bab1* and *bab2*, no Delta was observed at the plasma membrane, unlike in control TF cells (Fig 5C’, 5C”, 5D’ and 5D”). Instead, in anterior-most *hhG+* cells, Delta was detected only in its cytoplasmic vesicular form, while posterior-most *hhG+*/Tj+ cells did not present any Delta at the plasma membrane nor in vesicles as for most of the control CC population (Fig 5D’ and 5D”). Despite this, all medial *hhG+* cells strongly knocked down for *bab1* and *bab2* expressed the *E(spl)mβ-CD2* reporter indicative of active Notch signaling, as control niche cells (Fig 5E’, 5E”, 5F’ and 5F”). Therefore, strong reduction of Bab proteins in niche cells during larval stages did not prevent Notch signaling activation in these cells. This activation may be due to vesicular Delta present in the most anterior Tj-/*hhG+* cells (Fig 5D’ and 5D”) since they resemble control transition cells which have been shown to be Delta-sending cells presenting predominantly vesicular Delta and activating Notch signaling in adjacent CC precursors (Fig 5C’ and 5C” and [44]). Altogether, these results indicate that Bab1 and Bab2 are necessary for expression of some TF markers, which could be linked to the inability of Bab1 and Bab2 depleted *hhG+* cells to form proper TFs. On the other hand, Bab1 and Bab2 do not seem to be necessary for expression of four different CC markers. Therefore, the almost complete absence of GSC establishment next to *hhG+* cells deficient for Bab1 and Bab2 cannot be attributed to failure in CC specification as assessed by four different markers.

### Reduction of Bab1 and Bab2 levels specifically in GSC niche cells during larval stages leads to loss of expression of two *dpp* reporter transgenes in these cells

The absence of GSC establishment in the larval ovary upon efficient reduction of Bab proteins in niche cells could be due to a specific defect in *dpp* expression in CCs. Therefore, we tested whether *bab* gene functions control *dpp* expression in CCs using two transgenic lines reporting *dpp* expression. The first one, hereafter called *dpp-nlsGFP*, contains a construction with a large genomic region (about 44 kb) covering both the *dpp* regulatory and complete coding sequences into which a C-terminal GFP tag was introduced [58]. To test this line as a valid *dpp* reporter, we characterized its expression pattern in germaria of the adult ovary since endogenous *dpp* expression has been more clearly described at this stage than at the prepupal stage [13,15–17,19], and found that it reflects the endogenous *dpp* expression pattern in CCs and prefollicle cells (S6A Fig). The second transgene, *dppP4-lacZ*, contains a small region of *dpp* cis-regulatory sequences (1.5kb) that controls expression of the *lacZ* coding sequence [59]. This *dpp* transcriptional reporter was shown to be expressed in CCs, like endogenous *dpp*, but, unlike endogenous *dpp*, it was also expressed in TF cells and absent from prefollicle cells, thereby not fully recapitulating the endogenous *dpp* expression pattern.

**Fig 6:**
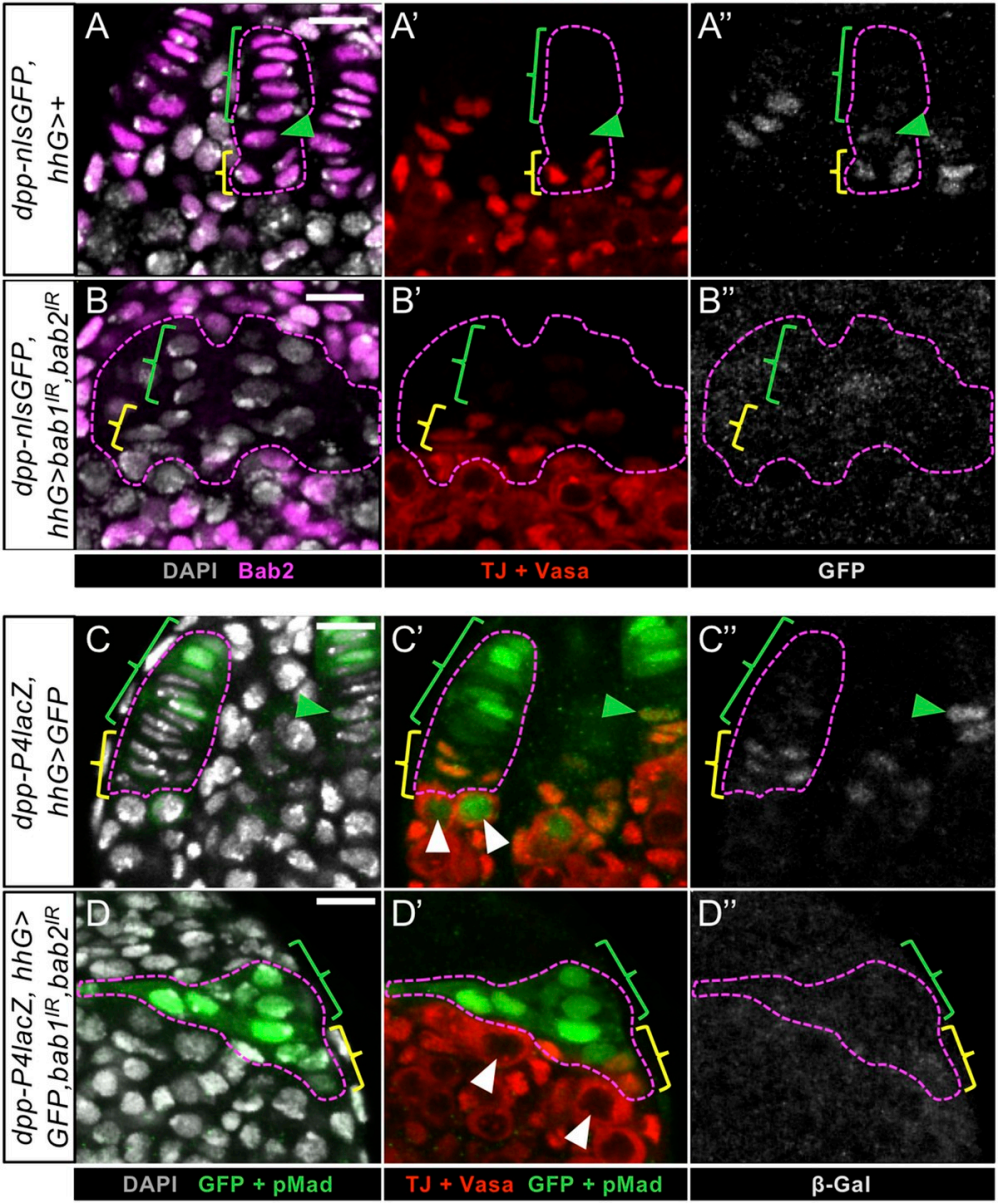
*bab1* and *bab2* functions in niche cells are required for expression of several *dpp* reporter transgenes. (A-D) Medial region of prepupal ovaries. Nuclei are labeled with DAPI (grey). Germ Cells (GCs) are visualized with immunostaining of cytoplasmic Vasa (red). Anterior is up. Scale bars: 10 μm. (A-A",C-C") One *hhG>GFP* medial niche is encircled (pink dotted line) in control ovaries and green and yellow brackets indicate Terminal Filament (TF) cells and Cap Cells (CCs), respectively. The green arrowheads point to transition cells. (B-B”, D-D”) The entire cluster of medial *hhG+* cells is encircled (pink dotted line) in *bab1* and *bab2* knockdown ovaries, and green and yellow brackets indicate the anterior- and posterior-most *hhG+* cells, respectively. (A,B) Somatic cells are marked with Bab2 (magenta). (A-A”) In a control ovary, *dpp-nlsGFP* (grey) is specifically expressed in CCs along with nuclear Traffic Jam (Tj, red) and sometimes in the transition cell, but not in TFs. (B-B”) Upon *bab1* and *bab2* RNAi-mediated knockdown, *dpp-nlsGFP* is not expressed in posterior-most *hhG+* cells that are positive for the nuclear CC marker Tj, and in contact with the GCs. (C-C”) In a control ovary, *dpp-P4lacZ* expression (grey) is found in CCs in contact with GSCs that are marked with nuclear pMad+ (green, white arrowheads) and often in the transition cell (green arrowhead). (D-D”) Upon *bab1* and *bab2* knockdown, *dpp-P4lacZ* expression is not detected in the posterior-most *hhG+* cells that are positive for the CC marker Tj, and this correlates with an absence of pMad in the underlying GCs (white arrowheads).

At the prepupal stage in the medial niche region of control ovaries containing either the *dpp-nlsGFP* or the *dpp-P4lacZ* transgene, GFP and β-Galactosidase (β-Gal) were detected in CCs (Fig 6A’, 6A”, 6C’ and 6C”). Dpp-nlsGFP was not found in TF cells, and rarely in transition cells (Fig 6A”), while β-Gal was slightly detectable in TF cells and often detected in transition cells (Fig 6C”). The CCs expressing the *dppP4-lacZ* transgene were in contact with GSCs (Fig 6C’). In striking contrast, upon efficient reduction of Bab1 and Bab2 in *hhG+* cells, *dpp-nlsGFP* and *dpp-P4lacZ* were not or only very faintly expressed in posterior most-*hhG+* cells of the medial region of the ovary considered as CC-like (Fig 6B–6B” and 6D–6D”) and this correlated with the absence of pMad in adjacent GCs (Fig 6B and 6D’). Altogether, these results indicate that, in niche cells, Bab1 and Bab2 are necessary for expression of two *dpp* transgenes in CCs at the prepupal stage.

### Reduction of Bab proteins in niche cells during larval stages leads to reduced levels of Engrailed/Invected in TF cells, but not in CCs

**Fig 7:**
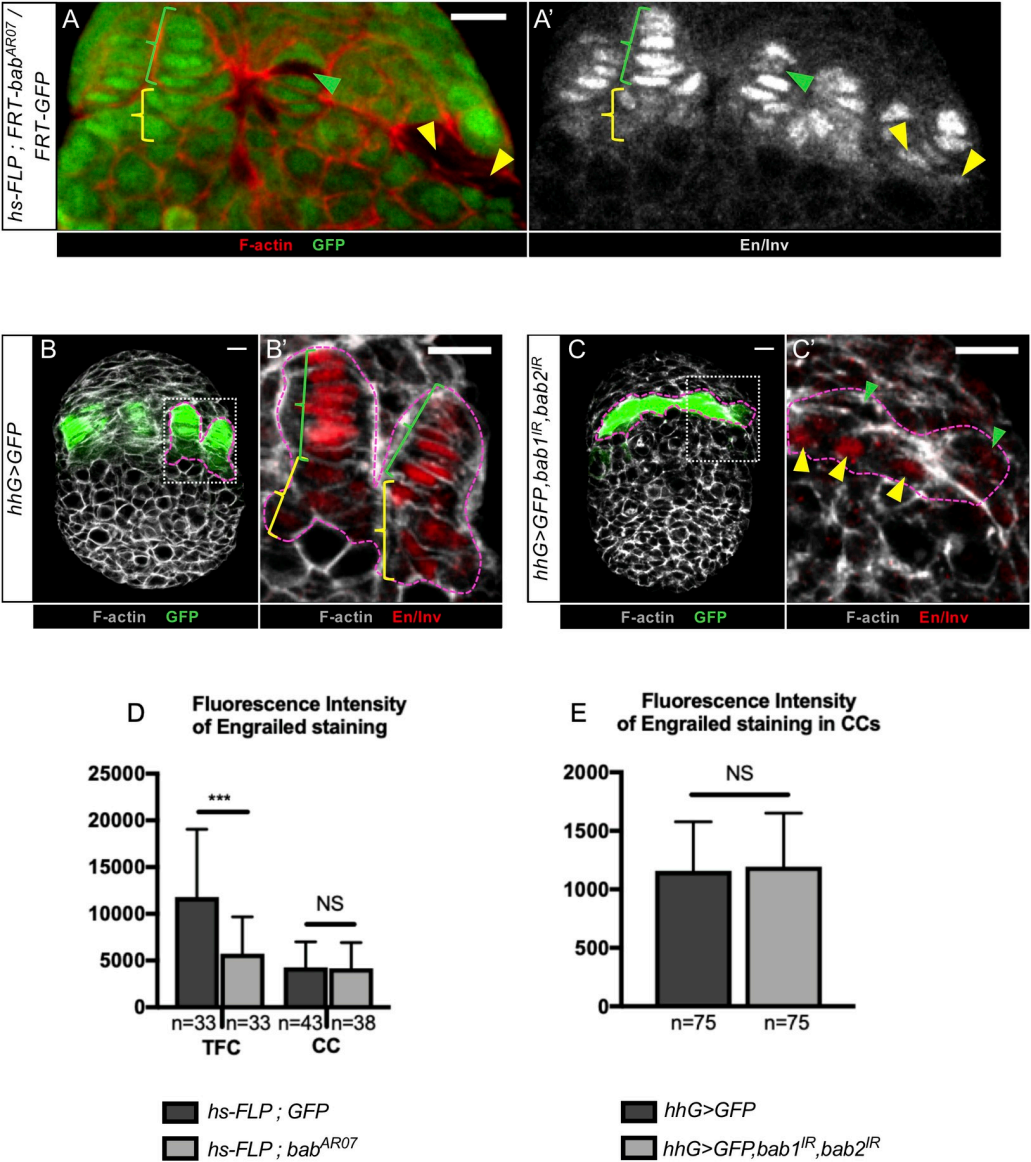
*bab* gene functions contribute to regulation of Engrailed/Invected accumulation in Terminal Filament cells but not in Cap Cells. (A-C') Prepupal ovaries immunostained for detection of GFP (green) and Engrailed/Invected (En/Inv) (grey in A’ and red in B’,C’). F-actin labeling is shown in red (A) and grey (B-C'). Anterior is up, medial is left. Scale bars: 10 μm. (A-A', B') Green and yellow brackets indicate Terminal Filament (TF) cells and Cap Cells (CCs), respectively. (C-C') The cluster of medial *hhG+* cells depleted of Bab1 and Bab2 is encircled (pink dotted line). Green and yellow arrowheads indicate the anterior- and posterior-most *hhG+* cells, respectively. (A-A’) Niche region of a mosaic ovary (*hs-FLP; FRT-bab*^*AR07*^*/FRT-GFP)* containing mitotic cell clones homozygous for the *bab*^*AR07*^ mutation that are marked by absence of GFP (green and yellow arrowheads). In *bab*^*AR07*^ mutant TF cells (green arrowheads), the signal for En/Inv is lower than that in wild type TF cells (green brackets). However, in *bab*^*AR07*^ mutant Cap Cells (CCs) in contact with GCs (yellow arrowheads), the level of En/Inv is similar to its endogenous level in wild type CCs (yellow bracket). (B,C) Prepupal ovaries expressing GFP in *hhG+* cells (*hhG>GFP)*, and in C, RNAi transgenes against *bab1* and *bab2 (hhG>GFP*, *bab1*^*IR*^, *bab2*^*IR*^) as well. (B’,C’) Higher magnifications of the regions framed with dotted lines in (B,C). The anterior-most cells *hhG+* cells of *hhG>GFP*, *bab1*^*IR*^, *bab2*^*IR*^ ovaries (C’, green arrowheads) present lower levels of En/Inv (C’, green arrowheads) than TF cells in the control (B’, green brackets). The posterior-most *hhG+* cells depleted of Bab1 and Bab2 (C', yellow arrowheads) show a similar level of En/Inv than in control CCs (B’, yellow brackets). (D) Graph comparing the fluorescence intensity (arbitrary units) of En/Inv in control niche and *bab*^*AR07*^ clonal cells. In *bab*^*AR07*^ mutant TF cells (*hs-FLP*, *bab*^*AR07*^), the En/Inv fluorescence intensity is more than 2-fold lower than in adjacent control TF cells (*hs-FLP*, *GFP*). However, in *bab*^*AR07*^ mutant CCs, the En/Inv fluorescence intensity is similar to that in adjacent control CCs. (E) Graph comparing the fluorescence intensity (arbitrary units) of En/Inv in CCs in the control (B', yellow brackets) and in posterior-most *hhG+* cells knocked down for *bab1* and *bab2* using RNAi (C', yellow arrowheads). Values are presented as means +s.d. p-values are calculated using a Kruskal-Wallis (D) or Mann-Whitney (E) test. n: sample size; NS: Not Significant (p>0.05); **** p<0.0001).

Like the *bab* genes, *engrailed* and its paralog *invected (en/inv)* have also been shown to be involved in proper TF formation [36]. In addition, evidence strongly suggests that En directly controls *dpp* expression in adult CCs [17]. In order to explore possible functional interactions between Bab and En/Inv proteins, we tested whether *en/inv* expression in niche cells of prepupal ovaries depends on Bab proteins. In control prepupal ovaries, we found that *en/inv* are specifically expressed in fully formed TFs and CCs (Fig 7A, 7A’, 7B and 7B’), as previously reported [31,40,41]. In addition, we observed a significant difference in En/Inv levels between TF cells and CCs (Fig 7A, 7A’, 7B and 7B’), with a three-fold higher level in control TF cells than in CCs (Fig 7D). Mitotic TF cell clones homozygous for *bab*^*AR07*^, a null allele for both *bab1* and *bab2* [34], showed a more than two-fold reduction in En/Inv levels when compared to control TF cells in the same prepupal ovaries (Fig 7A, 7A’ and 7D). In contrast, En/Inv levels in *bab*^*AR07*^ CC clones were not significantly different from those in wild-type CCs in the same ovary.

Using RNAi to knockdown *bab* gene functions in *hhG+* niche cells (Fig 7B–7C’), similar results were obtained. En/Inv levels were significantly lower in anterior-most *hhG+* cells in *hhG>GFP*, *bab1*^*IR*^, *bab2*^*IR*^ ovaries when compared to the *hhG>GFP* control TFs and CCs, while present at the same levels in posterior-most *hhG+* Bab depleted cells in contact with GCs (Fig 7B’, 7C’ and 7E). Taken together these results show that *bab* gene functions are required in TF cells to ensure high En/Inv accumulation at the prepupal stage but are not required in CCs to ensure normal low levels of En/Inv. Therefore, the function of Bab proteins for expression of *dpp* in CCs, as assayed with two *dpp* regulatory sequence-containing transgenes (see previous section), cannot be linked to En/Inv, since Bab proteins do not control En/Inv accumulation in these cells.

### Reduction of Engrailed/Invected levels in developing niches of larval ovaries does not prevent either TF formation or initial GSC establishment

**Fig 8:**
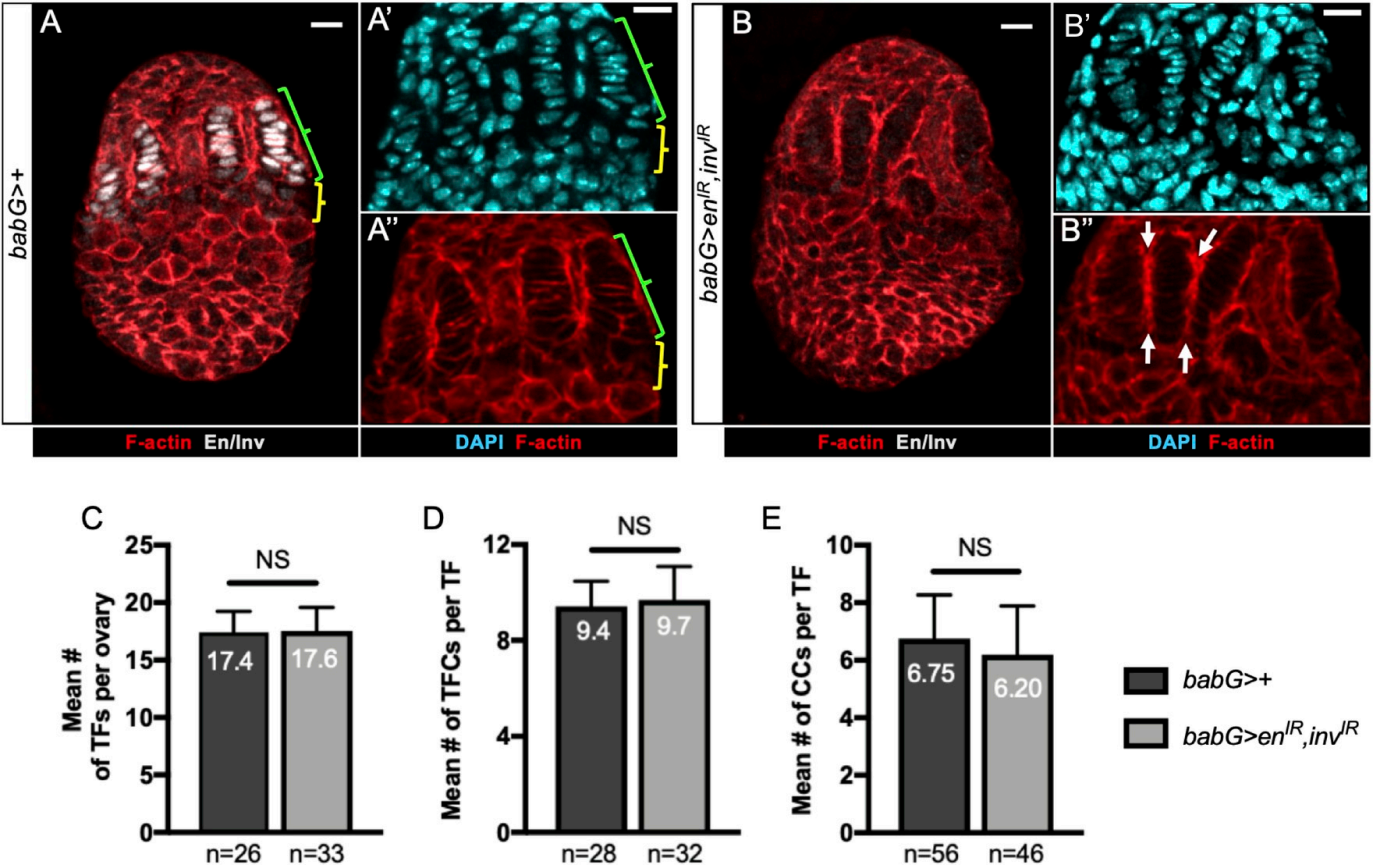
Reduction of Engrailed/Invected levels in developing niche cells does not impair Terminal Filament formation. (A-B”) Prepupal ovaries immunostained for detection of Engrailed/Invected (En/Inv) (grey). F-actin labeling is shown in red (A,A”,B,B”). Nuclei are labeled with DAPI (cyan). Anterior is up, medial is left. Scale bars: 10 μm. (A’,A”,B’,B”) Higher magnifications of the niche regions of the corresponding ovaries in (A,B). (A) Control ovary (*babG>*+), showing accumulation of En/Inv in the niche cells (Terminal Filament (TF) cells, green bracket and Cap Cells (CCs), yellow bracket). (B-B”) In an ovary carrying transgenes targeting *en/inv* for RNAi (*babG>UAS-en*^*IR*^, *UAS-inv*^*IR*^), En/Inv are efficiently depleted (B), while TF morphology resembles that of control TFs (B,B"). However, some TFs are not well separated from each other (arrows). (C-E) Graphs comparing different parameters related to TF formation, between control ovaries and ovaries depleted of En/Inv. (C) Mean number of TFs per ovary, (D) mean number of TF cells per TF and (E) mean number of CCs per TF. The TF cells were distinguished from CCs by their flattened nuclei and their flat shape, as determined by DAPI (A’,B’) and F-actin labeling (A",B"), respectively. CCs were distinguished from Intermingled Cells (ICs) using Bab1 immunostaining, which is high in CCs and low in ICs (Fig 1C’). None of these parameters were significantly different between control and En/Inv depleted ovaries. Values are presented as means +s.d., p-values are calculated using a two-tailed t-test (C, E) or a Mann-Whitney test (D). n: sample size; NS: Not Significant (p>0.05).

To explore the implication of *en/inv* in niche formation using the same experimental approaches as for *bab*, we tested whether knockdown of *en/inv* in all niche cells would phenocopy the defect in prepupal TF morphogenesis provoked upon strong reduction of Bab1 and Bab2 in these same cells. To achieve efficient reduction of *en/inv*, we used the *babG* driver and RNAi transgenes directed against *en* and *inv* (*babG>en*^*IR*^,*inv*^*IR*^). Although this led to undetectable En/Inv in niche cells (Fig 8A and 8B), no abnormalities in TF formation were observed (Fig 8A–8E). The only mutant phenotype observed in *babG>en*^*IR*^,*inv*^*IR*^ ovaries was a defect in TF individualization (Fig 8B–8B”), normally resulting from the migration of anterior somatic cells between TF stacks. These results strongly suggest that *en/inv* function is not essential for TF formation. Interestingly, these results also indicate that the function of *bab* in TF formation most likely does not depend on *en/inv* function.

**Fig 9:**
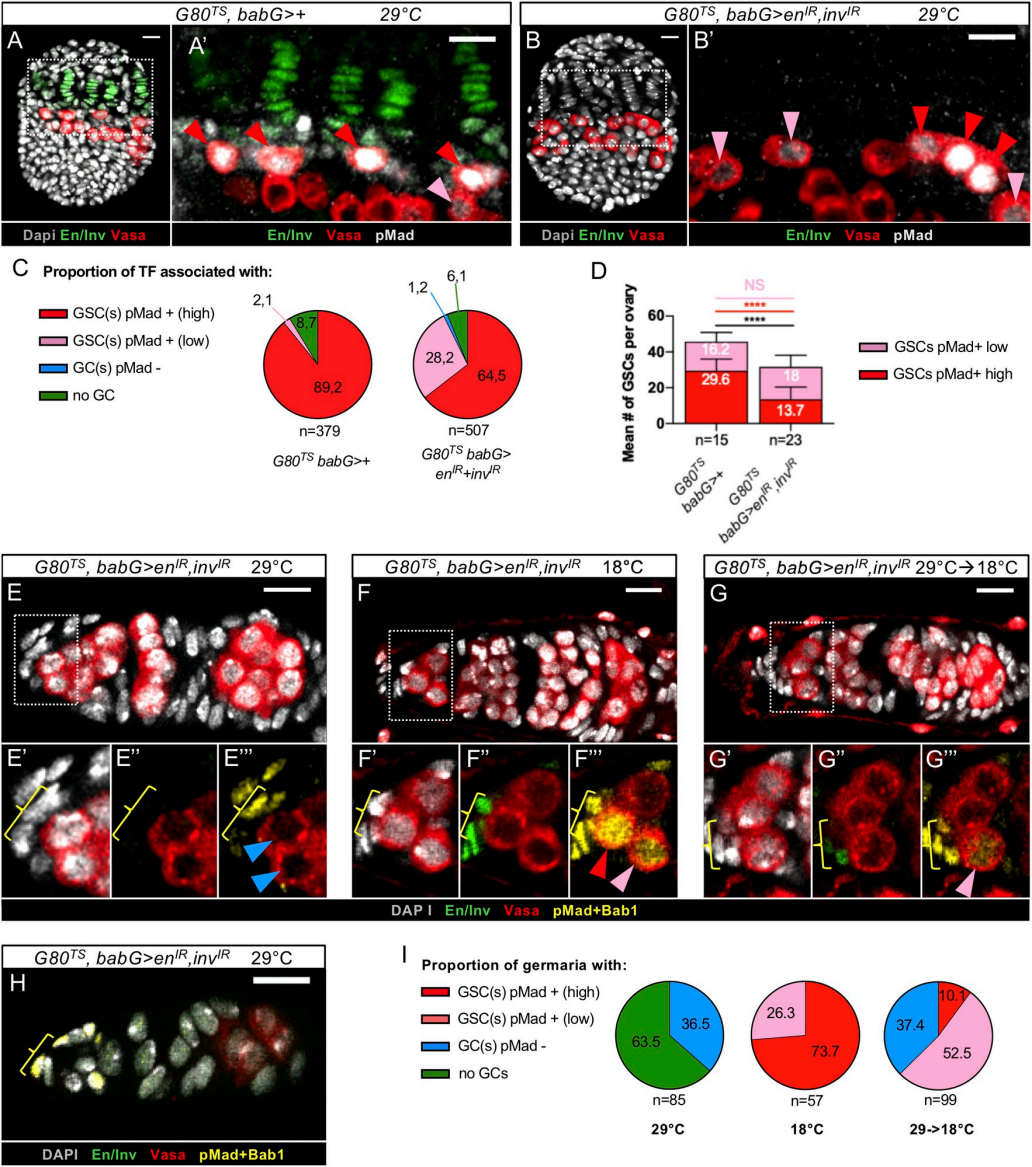
Reduction of *en/inv* functions during larval stages leads to only a partial loss of Germline Stem Cells by the prepupal stage but to strong Germline Stem Cells loss in adult germaria. (A-B) Prepupal ovaries immunostained for detection of Engrailed/Invected (En/Inv) (green), Vasa (red) and pMad (grey). Nuclei are labeled with DAPI (grey). Anterior is up, medial is left. Scale bars: 10 μm. (A’,B’) Higher magnifications of the niche regions of the corresponding ovaries in (A,B). In control ovaries (*G80*^*TS*^*; babG>+*) and ovaries depleted of En/Inv *(G80*^*TS*^*; babG/UAS-en*^*IR*^, *UAS-inv*^*IR*^*)*, Germline Stem Cells (GSCs) with both high and low pMad levels are present (red and pink arrowheads, respectively). (C) Pie charts comparing the proportion of prepupal niches associated with: at least one GSC with a high level of pMad (red), at least one GSC with a low level of pMad (pink), GCs without nuclear pMad (blue) and no Germline Cells (GCs) (green). The depletion of En/Inv does not change the proportion of prepupal niches associated with at least one GSC (sum of pink and red portions, 91.3% and 92.7% in control and En/Inv-depleted niches, respectively). (D) Graph comparing the mean number of GSCs per prepupal ovary in control and En/Inv depleted ovaries. The number of GSCs per ovary is significantly lower in En/Inv depleted ovaries (black bar), due to the significantly lower mean number of high-level pMad+ GSCs (red bar) and not to the mean number of low-level pMad+ GSCs (pink bar). Values are presented as means +s.d. p-values are calculated using a two-tailed t-test or a Mann-Whitney test. n: sample size; NS: Not Significant (p>0.05); **** (p<0.0001). (E-H) Germaria from 1 day-old adult females carrying transgenes for temperature-controlled RNAi of *en/inv* (*G80*^*TS*^*; babG/UAS-en*^*IR*^, *UAS-inv*^*IR*^*)*, immunostained for En/Inv (green), Vasa (red) and pMad and Bab1 (yellow). Anterior is up. Scale bars: 10μm. The temperatures used for raising the flies are indicated on top of the images. (E’-E”’,F’-F”’, G’-G”’) Higher magnifications of the corresponding niche regions in (E,F,G). (E-E‴) Germarium of a female raised at 29°C from the L1 stage to one-day old adulthood showing efficient depletion of En/Inv in Cap Cells (CCs) (9E”, yellow bracket). This germarium does not contain any GSCs (E”’, blue arrowheads). (F-F‴) Germarium of a female raised at 18°C from the L1 stage to one-day old adulthood is not depleted of En/Inv in CCs (F”, yellow bracket) and exhibits GSCs with high and low levels of pMad (F”’, red and pink arrowheads, respectively). (G-G‴) Germarium of a female raised at 29°C from L1 stage and transferred to 18°C at the prepupal stage, showing the presence of En/Inv in CCs (G”, yellow bracket) and a GSC (G”’, pink arrowhead). (H) Germarium of a female raised at 29°C from the L1 stage to one-day old adulthood showing efficient depletion of En/Inv in CCs (yellow bracket) but no GCs close to the niche. (I) Pie charts comparing the proportion of germaria present in ovaries from females raised at the indicated temperatures with: at least one GSC with a high level of pMad (red), at least one GSC with a low level of pMad (pink), GCs without nuclear pMad (blue), or no GCs (green). The re-expression of en*/inv* in ovaries from the prepupal stage onwards (29->18°C) leads to significant rescue (from 0% to 62.6%) of the proportion of germaria containing GSCs.

Since in adult ovary GSC niches *en* is known to be necessary for GSC maintenance through the direct regulation of *dpp* expression in CCs [17,23], we tested whether *en/inv* gene functions are also necessary for GSC establishment in ovaries by the prepupal stage. Upon *env/inv* knockdown throughout larval development, prepupal ovaries displayed niches with high and low pMad levels in GSCs as in control (Fig 9A’ and 9B’), with a normal proportion of TFs associated with GSCs (Fig 9C). The average number of GSCs per ovary, however, was about 30% lower in *en/inv* RNAi ovaries than in control ovaries (Fig 9D). This difference corresponds to a lower proportion of high pMad+ GSCs per ovary (about half as many, Fig 9D). It is possible that under RNAi conditions enough En/Inv is present during larval ovary development to contribute to GSC establishment. However, the phenotype obtained is much weaker than that using RNAi to deplete Bab1 and Bab2 (Fig 3F). Therefore, it is possible to propose that GSCs with high pMad levels may be normally recruited at the end of the L3 stage in En/Inv RNAi-depleted niches, but that these niches may not have the capacity to maintain GSC status efficiently up to the prepupal stage. In order to assess whether GSC maintenance depends on *en/inv* function as of the prepupal stage, we used the *Gal80*^*TS*^ system (hereafter named *G80*^*TS*^), which allows Gal4 activity at 29°C and impedes it at 18°C. We tested the fate of GSCs in 1-day-old adult females of the same genotype (*G80*^*TS*^, *babG> en*^*IR*^, *inv*^IR^) but raised at different temperatures (Fig 9E–9G). Using 18°C as the rearing temperature, Env/Inv were present normally in niches (Fig 9F–9F”) and GSCs were present in every germaria (Fig 9F”’ and 9I). In contrast, GSC niches of females maintained at 29°C throughout development were efficiently knocked down for *en/inv* (Fig 9E–9E”) and 100% of corresponding germaria lacked GSCs (Fig 9E”’ and 9I), with a majority of germaria even devoid of any GCs (Fig 9H and 9I). Thus, all newly established GSCs in prepupal ovaries depleted of En/Inv were no longer present at adulthood. This result is consistent with the hypothesis that when En/Inv are efficiently reduced in larval stages, GSC establishment occurs but GSCs are not maintained if En/Inv continues to be reduced. This is further supported by the fact that partial re-expression of *en/inv* in niche cells from the onset of pupariation (29°C to 18°C switch) substantially rescued the adult loss of GSCs phenotype from 0% to more than 60% GSCs present (Fig 9G–9G”’ and 9I). Altogether, these results strongly suggest that *en/inv* functions are not essential for initial GSC establishment within newly formed niches, but would be essential for GSC maintenance in niches starting from at least the beginning of the pupal stage, and possibly even earlier, as soon as GSCs are established in larval stages.

### Knockdown of *bab* genes in the adult ovary extinguishes *dpp* transgene expression and reduces GSC number

**Fig 10:**
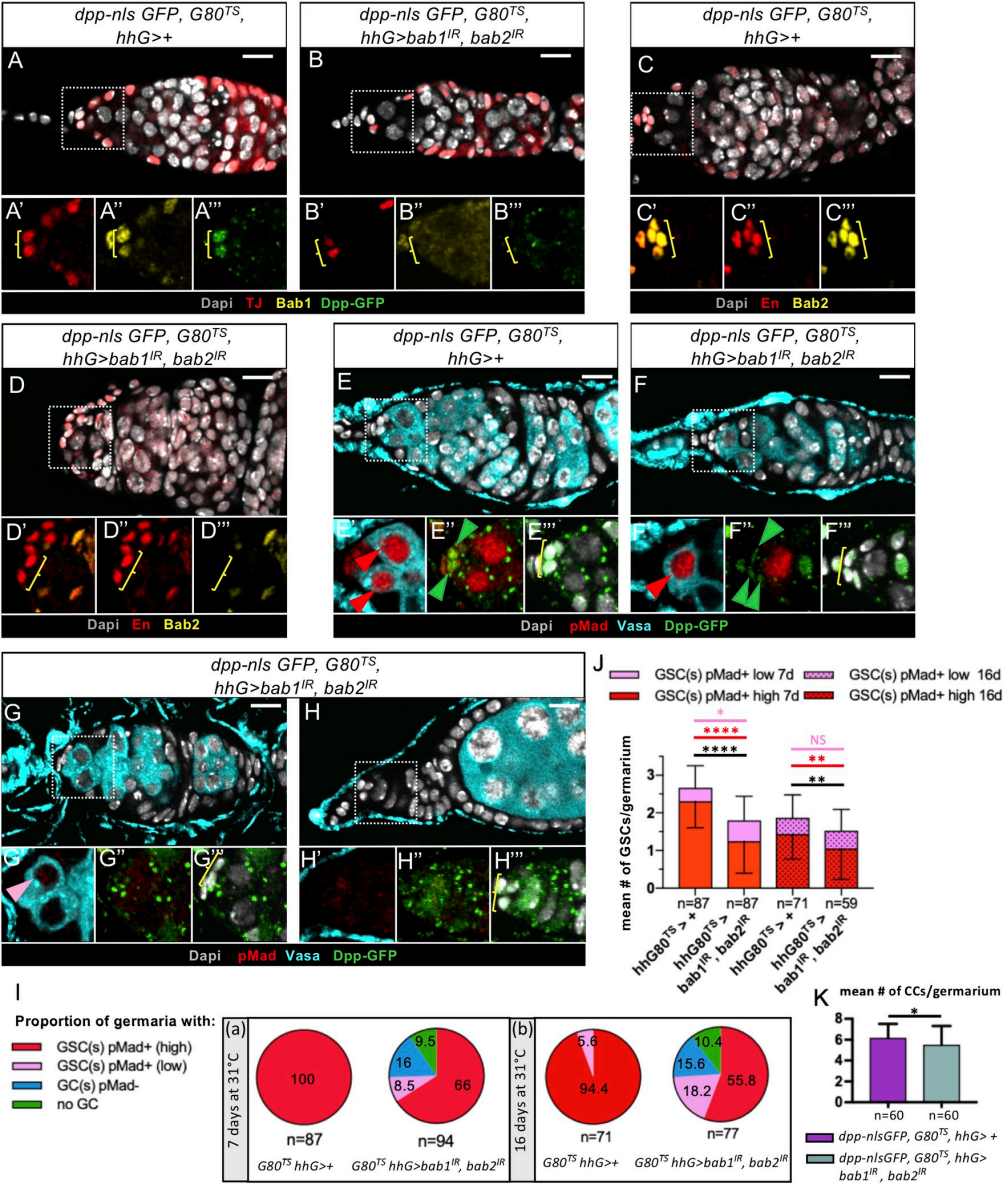
Bab proteins are required for the activation of a transgene reporting *dpp* expression in Cap Cells and contribute to GSC maintenance in adult ovaries. (A-H) Adult germaria from females carrying transgenes for temperature-controlled RNAi of *bab1* and *bab2* (*G80*^*TS*^*; hhG/UAS-bab1*^*IR*^, *UAS-bab2*^*IR*^) or of the control genotype (*G80*^*TS*^*; hhG/+*). Females were raised at 18°C to the young pupal stage, shifted to 29°C until eclosion and shifted to 31°C for 7 days. Ovaries were immunostained for detection of Dpp-nlsGFP (green), Traffic Jam (Tj, red) and Bab1 (yellow) (A-B), Engrailed/Invected (En/Inv, red) and Bab2 (yellow) (C-D), and pMad (red) and Vasa (cyan) (E-G). Nuclei are labeled with DAPI (grey). (A’-A”’ to H’-H”’) Higher magnifications of the corresponding niche regions are shown (dotted lines). Anterior is to the left. Scale bars: 10μm. Yellow brackets indicate CCs, detected according to their location in the germarium and to their expression of CC markers, Tj (A-B) and En/Inv (C-D). (A-D) Germaria showing efficient depletion of Bab1 (B”) and Bab2 (D”’) in Cap Cells (CCs), correlated with barely detectable expression of *dpp-nlsGFP* in CCs (B”’) when compared to the corresponding control (A”, C”’ and A”’, respectively). (E-H”’) Niches depleted of Bab1 and Bab2 are associated with GSCs exhibiting high to barely detectable levels of pMad (F’,G’, red and pink arrowheads, respectively, compared to the control, E’). Germaria that do not contain any GCs close to the niche are also observed (H’). The expression of *dpp-nlsGFP* (E”,F”, green arrowheads) can be detected at a very low level in some CCs (F”) compared to the control (E”), but is undetectable in the majority of cases (G”). (I) Pie charts comparing the proportion of germaria from ovaries of control and *bab* gene knockdown females with: at least one GSC with a high level of pMad (red), at least one GSC with a low level of pMad (pink), GCs without nuclear pMad (blue), or no GCs (green). The knockdown of *bab* genes thus leads to the appearance of germaria devoid of GSCs and to rudimentary germaria devoid of GCs. (J) Graph comparing the mean number of GSCs per germarium in control ovaries and in ovaries depleted of Bab proteins from 7- or 16-day old females. At 7 days, the average number of GSC per germaria is significantly lower (black bar) in Bab depleted ovaries (1.8) compared to the controls (2.7), largely due to the significantly lower number of high-level pMad+ GSCs (red bar). At 16 days, similar results are obtained with the exception that no difference is observed for low-level pMad+ GSCs (pink bar). Values are presented as means +s.d., p-values are calculated using a two-tailed t-test or a Mann-Whitney test. n: sample size; NS: Not Significant (p>0.05); * (p<0.05); **** (p<0.0001). (K) Graph comparing the mean number of CCs per germarium in control germaria (*dpp-nlsGFP*, *G80*^*TS*^, *hhG>+*) to that in germaria with temporally controlled RNAi-induced knockdown of *bab* genes from the young pupal stage onwards (*G80*^*TS*^*; hhG/UAS-bab1*^*IR*^, *UAS-bab2*^*IR*^). A small but significantly lower number of CCs/germarium was observed in *bab1* and *bab2* knockdown adult ovaries when compared to the control. Values are presented as means +s.d. p-values are calculated using a two-tailed t-test with Welch’s correction. n: sample size; * (p<0.05).

We next tested whether, beyond their function in GSC niches during larval stages, Bab proteins also play a role in adult GSC niches. In order to knockdown *bab* genes in niche cells only during the adult stage, we used the *G80*^*TS*^ system and analyzed ovaries from 7-day-old females. To set up the experimental design, we raised *G80*^*TS*^, *hhG>UAS-GFP* flies at 18°C until pupal stage and shifted to the restrictive temperature (29°C, 31°C) at different stages. We found that proper reactivation of *hhG* in adult ovaries occurred when these flies were shifted to 29°C as of 24h after puparium formation (a stage where GSC niches are already formed and GSCs already recruited), followed by a shift to 31°C upon eclosion (S7A–S7A” and S7B–S7B” Fig). With these experimental conditions, adult ovaries of *G80*^*TS*^,*hhG>bab2*^*IR*^ 7-day old females, though displaying a strong reduction of Bab2 levels in niche cells (S8A–S8B”’ Fig), were normal regarding the structure of the germarium and the mean number of GSCs per germarium (S8C–S8E Fig). For Bab1, it has been reported that adult ovaries homozygous for the strong hypomorphic *bab1*^*A128*^ allele were normal [34]. Together, these results indicate that the depletion of Bab1 throughout development and in the adult or of Bab2 only as of the pupal stage does not affect adult niche formation and function. In contrast, in adult ovaries from *G80*^*TS*^, *bab1*^*A128*^, *hhG>bab2*^*IR*^ females, although a few normal germaria and ovarioles were present, the majority of the ovaries were rudimentary and largely devoid of clearly distinguishable ovarioles and GCs (S7C–S7F” Fig). In contrast, prepupal ovaries from females of the same genotype reared at 18°C, that displayed normal levels of Bab2 and no detectable Bab1, presented a normal morphology and niches containing GSCs (S7G–S7G" and S7J–S7J” Fig). Therefore, efficient reduction of Bab1 throughout development, along with depletion of Bab2 only as of the pupal stage, leads to loss of GSCs that were initially present at the prepupal stage. This mutant phenotype was too strong to allow analysis of possible defects in GSC maintenance and *dpp* transgene expression specifically. Thus, we used the protocol described above to reduce both Bab1 and Bab2 levels with RNAi constructs only as of pupal stages and were able to recover less altered ovaries. In adult germaria of *G80*^*TS*^; *hhG>bab1*^*IR*^,*bab2*^*IR*^ females also carrying the *dpp-nlsGFP* transgene, Bab1 and Bab2 were undetectable in CCs (Fig 10A–10D”) and strikingly, these CCs were also almost or completely devoid of Dpp-nlsGFP signal (Fig 10A”’, 10B”’, 10E”, 10E”’, 10F”, 10F”’, 10G”, 10G”’, 10H” and 10H”’). Therefore, in adult ovaries, Bab proteins are necessary for expression of this *dpp* transgene in CCs, as we showed is also the case in larval ovaries. Furthermore, in these adult ovaries, only 74.5% of germaria had at least one GSCs (Fig 10E–10F”’ and 10I(a)), while other germaria had either GCs with no detectable pMad in the niche region (Fig 10G–10G”’ and 10I(a)) or no GCs at all (Fig 10H–10H”’ and 10I(a)). When GSCs were present, their average number was significantly lower than in control adult ovaries (Fig 10J). Therefore, Bab proteins are important in CCs specifically during pupal and/or adult stages for GSC maintenance. Adult niches depleted of Bab1 and Bab2 and containing GCs displayed an overall normal structure of the anterior part of the germarium, with the presence of TF cells and CCs, although a small decrease in CC number was observed (Fig 10K).

To determine whether loss of GSCs in the adult ovary was progressive upon Bab protein depletion, we performed the analysis on the same *G80*^*TS*^; *hhG>bab1*^*IR*^,*bab2*^*IR*^ females but at 16-days of age. We observed a decline with age in the average number of GSCs in control females, as previously described [60], associated with a decrease in the mean number of GSCs with high pMad levels (Fig 10J). There was a smaller, but nonetheless significant, difference in GSC number between control and *bab* knockdown conditions in germaria at 16 days when compared to that of 7-day old females. However, the proportion of germaria with at least one GSC did not decrease between 7 and 16 days (Fig 10I(a,b)). Altogether, these results strongly suggest that Bab proteins are implicated in GSC maintenance through positive control of *dpp* expression in CCs and, possibly also through a small effect on establishing a full set of CCs per niche.

### Overexpression of *bab2* in germaria leads to GSC-like tumors

Since Bab proteins are necessary for TF morphogenesis and initial GSC establishment, we then asked whether these proteins could be sufficient to induce formation of ectopic TFs and/or GSCs. With this aim, we expressed *UAS-bab1* or *UAS-bab2* constructs with the *C587-Gal4* driver (called *C587G* hereafter) at either 25°C or 29°C. *C587G* is expressed in most, but not all, somatic cells of developing female gonads [32]. We obtained prepupal ovaries exhibiting higher levels of Bab1 or Bab2 than in control ovaries, particularly in ICs, and ectopic Bab1 in basal somatic cells (S9A–S9C’ Fig). We found that ICs with higher Bab1 or Bab2 levels than in the control did not exhibit ectopic En/Inv accumulation (S9A”, S9B” and S9C” Fig). In addition, we did not detect any obvious change in Bab1 levels in cells overexpressing Bab2, and reciprocally (S9B, S9B’, S9C and S9C’ Fig). Finally, neither of these conditions appeared to affect the organization of pupal ovaries.

**Fig 11:**
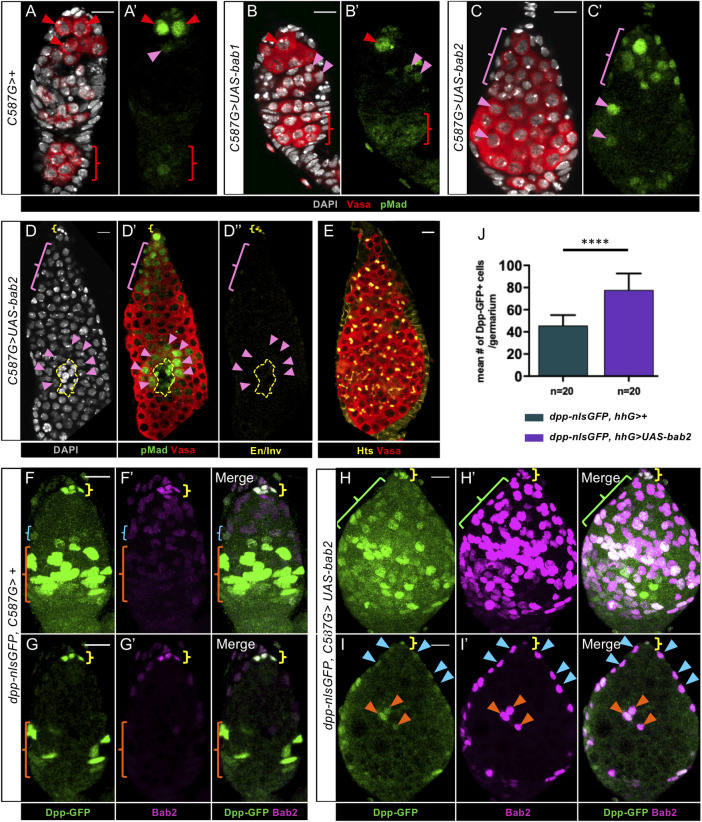
The overexpression of *bab2* in somatic cells outside the niche is sufficient to induce GSC expansion in adult germaria and ectopic expression of a *dpp-nlsGFP* reporter. (A-I) Adult germaria from 10-day old females raised at 18°C during development and transferred to 29°C upon eclosion. DAPI nuclear labeling is in grey. Yellow brackets indicate the GSC niches. Anterior is up. Scale bars: 10μm. (A-D) Ovaries were immunostained for detection of pMad (green) and Vasa (red). (A,A') In a control *C578G>+* ovary, Germline Stem Cells (GSCs) are marked with pMad at high or low levels (red and pink arrowheads, respectively). pMad also accumulates faintly in the maturating germline cyst (red brackets). (B,B’) In rare cases, the ectopic expression of *bab1* (*C578>UAS-bab1*) leads to the presence of ectopic pMad+ Germ Cells (GCs) (pink arrowheads). (C-C’) Inducing higher levels of Bab2 (*C578>UAS-bab2*) in ECs and prefollicle cells leads to an increase in the number of GSCs in continuity with the niche (pink bracket) or at ectopic positions in the germaria (pink arrowheads). (D-D”) Engrailed/Invected (En/Inv, yellow) are not detected in somatic cells (yellow dotted lines) in contact with the ectopic GSCs (pink arrowheads) contrasting with the accumulation of these proteins in the endogenous niche (yellow bracket). (E) The overexpression of *bab2* leads to the formation of huge germaria with GSC-like tumors marked by the presence of spectrosome-containing (Hts, yellow) GCs (Vasa, red). (F-F’, H-H’) Projections of all confocal sections, (G-G’, I-I’) projections of 4 adjacent confocal sections. (F-I) Ovaries were immunostained for detection of Dpp-nlsGFP (green) and Bab2 (magenta). (F,G) In the germaria of a control ovary from a female carrying the *dpp-nlsGFP* transgene (*dpp-nlsGFP*, *C587G > +*), GFP is present at high levels in Cap Cells (CCs; yellow brackets), and in prefollicle cells (orange brackets), while low GFP levels are observed in posterior Escort Cells (ECs, F, blue brackets). (F’, G’) Bab2 accumulates at high levels in CCs (yellow brackets), at low levels in more anterior ECs (below yellow brackets through blue bracket region). Barely detectable levels of Bab2 are present in prefollicle cells (orange brackets). Dpp-nlsGFP and Bab2 are therefore largely mutually exclusive except in niche cells. (F-I’) Upon induction of increased levels of Bab2 (*dpp-nlsGFP*, *C587G>UAS-bab2*), low ectopic Dpp-nlsGFP signal is present in some ECs (H-H’ green bracket, I-I’ blue arrowheads, and corresponding merges), and higher Dpp-nlsGFP signal is found in more posterior somatic cells (H-H’ below green bracket, I-I’ orange arrowheads, and corresponding merges), whereas this is not the case in the control (*dpp-nlsGFP*, *C587G*>+, F-G’ and merges). (J) Graph comparing the mean number of Dpp-nlsGFP+ cells in control and in Bab2-overexpressing germaria. When females raised at 18°C during development were transferred to either 29°C or 31°C for 10 days upon eclosion, a mean of 78.0 Dpp-nlsGFP-positive cells per germarium was obtained with Bab2 overexpression when compared to a mean of 45.7 GFP-positive cells per control germarium. No statistical difference was obtained between shifts at 29°C or 31°C. Values are presented as mean +s.d., p-values are calculated using a two-tailed t-test. n = sample size; **** (p<0.0001).

In adult germaria, *C587G* has been shown to be expressed only in ECs and early prefollicle cells [18]. In order to test the effect of expressing *bab1* ectopically or over-expressing *bab2* in adult ovaries, we analyzed ovaries from 10-day old females raised at 29°C as of eclosion. Under these conditions, Bab1 and Bab2 accumulated strongly in ECs and prefollicle cells (S10 Fig and Fig 11H' and 11I'). We did not observe any cross-regulation between the two genes (S10 Fig). Strikingly, in ovaries overexpressing *bab2*, all germaria contained more than the normal number of GSCs, some in continuity with the niche and others at ectopic locations (Fig 11A, 11A’, 11C, 11C’ and 11D). Among these germaria, 74% exhibited more than 20 GSCs and 26% between 5 and 20 GSCs (n = 137), when compared to 0 and 13% (n = 144), respectively, for control germaria (S1 Dataset). In many cases, overexpression of *bab2* led to the formation of huge germaria with GSC-like tumors as marked by the presence of spectrosomes (Hts+ spherical cytoplasmic structures, Fig 11E). In these tumorous germaria, no ectopic En/Inv was detected in somatic cells outside of the endogenous niche (Fig 11D’). Upon ectopic expression of *bab1*, only 4 germaria out of 133 exhibited ectopic GCs in which the BMP pathway was activated (Fig 11B and 11B’), suggesting that Bab1 does not have the equivalent capacity as Bab2, or is not expressed at a high enough level in the condition tested to affect GSC number significantly. Altogether, these results show that *bab2* overexpression in somatic cells outside of the adult GSC niche is sufficient to induce activation of the BMP pathway in adjacent GCs, thereby producing numerous ectopic GSCs and tumorous germaria.

Since Dpp is known to be one of the niche associated signals promoting GSC self-renewal, we next investigated whether *bab2* overexpression in somatic cells outside of the adult GSC niche could induce expression of *dpp* reporters. We did not detect any ectopic *lacZ* expression when using *dppP4-lacZ*, which, although expressed in adult CCs like *dpp*, does not faithfully reproduce endogenous *dpp* expression as it is expressed in TF cells and not in prefollicle cells [13]. On the other hand, presence of the Dpp-nlsGFP fusion protein reflects endogenous *dpp* expression in the adult ovary, as it is found in CCs and prefollicle cells and not in TF cells (S6 Fig and Fig 11F and 11G) [13,15–17,19]. Upon overexpression of Bab2, Dpp-nlsGFP was found to be present ectopically in somatic cells between the GSC niche and the prefollicle cells along with Bab2 (Fig 11F–11I’ and corresponding merges). In addition, towards the middle of the germaria, somatic cells with both high Dpp-nlsGFP and Bab2 seemed to be more numerous than in the control (Fig 11F–11I’). Quantification showed that germaria of females overexpressing *bab2* contained significantly more GFP-positive cells than control germaria (Fig 11J). Together, these results indicate that Bab2 somatic overexpression in adult germaria leads to expanded expression of a *dpp-GFP* genomic transgene and production of tumors of GSC-like cells in which the BMP/Dpp pathway is activated, independently of *En/Inv* expression.

## Discussion

### The presence of either Bab1 or Bab2 in precursor niche cells during larval ovary development is necessary for functional GSC niche formation

The two *bab* paralogs encode transcription factors that have been shown to be required for ovary morphogenesis, leg proximal-distal differentiation, and sexually-dimorphic abdomen pigmentation [26,27,29,52,61]. *bab1* and *bab2* display at least partially overlapping expression patterns in these three organs [34,61,62]. Indeed, Bab1 is always present with Bab2, while Bab2 is present alone in additional territories in the leg and ovary. The question of the individual functions between Bab paralogs has been hampered in part by their overlapping expression (at least during leg and ovary development), but especially by the lack of any known *bab2* null mutation not affecting *bab1*. For *bab1*, the *bab*^*A128*^ allele has been shown to be associated with loss of detectable Bab1 protein without affecting Bab2 levels (confirmed in the ovary in the present study), but no equivalent allele has been characterized for *bab2* [34,63]. Roeske and co-workers [53] addressed the question of individual necessities of *bab1* and *bab2* in sex-specific abdomen pigmentation using tissue-specific expression of *bab1* and *bab2* shmiR constructs. They showed that the presence of both *bab1* and *bab2* (therefore four doses of Bab proteins) is necessary for efficient repression of pigmentation of female abdomen segments A5 and A6. In addition, their study and that of Couderc and co-workers [34] indicate equivalent capacities for Bab1 and Bab2 in this system since ectopic expression of either one at the same level leads to a similar decrease in pigmentation of segments A5 and A6 in males. Taken together, these results suggest functional equivalence between Bab1 and Bab2 proteins for sexually-dimorphic abdominal pigmentation and indicate that this process is particularly sensitive to Bab protein levels. Finally, one of the only direct targets for Bab protein transcriptional regulation, the *yellow* gene, has been identified in this system [53].

Our results indicate that efficient depletion of either Bab1 or Bab2 in developing ovary niche cells during larval stages leads to formation of normal TFs and correct establishment of initial GSCs by the prepupal stage. However, we cannot exclude that use of a strong hypomorphic *bab1* allele (*bab*^*A128*^) or RNAi-mediated depletion of *bab1* or *bab2*, which lead to undetectable levels of each protein in developing GSC niches, nonetheless allows enough remaining Bab1 or Bab2 to carry out their functions in these two processes. On the other hand, efficiently reducing both Bab proteins at the same time using the same tools led to very dramatic defects in TF formation and GSC recruitment. Therefore, in larval ovaries, two normal doses of either Bab1 or Bab2 proteins are sufficient for efficient TF formation and GSC establishment. In addition, in double heterozygous females for amorphic or strong hypomorphic *bab1* and *bab2* alleles, we have previously shown that TF formation occurs and that an excess in TF number is even produced [29]. Therefore, one dose of each of Bab1 and Bab2 allows for TF formation. In contrast, ectopic expression or overexpression of *bab1* or *bab2*, does not always produce the same effects. In this study, we found that somatic overexpression of *bab2* in germaria led to production of GSC-like tumors, while under similar conditions *bab1* ectopic expression did not affect GSC number significantly. Thus, either Bab1 does not have the equivalent capacity as Bab2, or it is not expressed at a high enough level, perhaps because of a difference in the expression levels of the *UAS-bab1* and *UAS-bab2* transgenes. In other contexts, Bab protein activities do not seem fully equivalent. In fact, *bab* mutant rescue experiments conducted with respect to leg and ovary developmental mutant phenotypes have shown that expression of either *bab1* or *bab2* is able to rescue the mutant phenotypes, but that Bab2 does so more efficiently [64]. In addition, *bab2* has a much larger expression domain than *bab1* in particular in the ovary, and removing *bab2* from both GSC niches and the rest of the somatic ovarian cells leads to a much stronger phenotype of atrophied ovaries than removing *bab2* only from niche cells precursors ([34] and this study). Thus, the role of Bab proteins in different tissues seems to require various doses of these proteins suggesting that they may have specific transcriptional targets in different tissues.

### Newly identified roles for Bab proteins in niches for *dpp* reporter expression in Cap Cells and initial establishment of adjacent GSCs

The BMP family member Dpp, known to be emitted principally by CCs within germarial niches, has been shown to be essential for maintenance of GSC status in the adult ovary [8,17,18]. During the larval to pupal transition, Dpp produced by niches has also been shown to be required for GSC establishment [32,45]. We showed that when both *bab1* and *bab2* functions were removed from precursor niche cells during larval development, these cells were not organized into TFs and GSC establishment, as evidenced by activated Dpp signal transduction, was largely absent by the prepupal stage.

Using several markers, we showed that TF cell specification seemed particularly perturbed when Bab1 and Bab2 were both efficiently depleted in niche cell precursors. Though Tj expression was absent as in control TF cells, En/Inv levels were two-fold lower, and *P1444-lacZ* expression and membranous Delta were undetectable unlike in control TF cells. It is therefore not possible to attribute a clear identity to these cells. Incorrect TF cell specification could explain why these cells are unable to undergo proper TF morphogenesis. In contrast, CC specification was not affected according to the expression pattern of the same markers in Bab1 and Bab2-depleted developing niche cells suggesting that several aspects of this process do not depend on *bab* gene functions.

However, we found that two transgenes containing *dpp* transcriptional regulatory sequences were expressed normally in control CCs, but were not, or only very faintly, expressed in *bab* deficient CCs in prepupae and adult ovaries. Since *hhG+* niche cells with undetectable levels of Bab proteins express some CC markers but not these *dpp* transgenes, these *hhG+* cells are partially defective CCs. These results indicate that Bab proteins may thus be necessary for normal *dpp* expression in CCs, which is essential for the function of these cells in GSC establishment [32,45]. The control of *dpp* expression by Bab proteins may be direct or indirect. One of the *dpp* expression reporters used, *dppP4-lacZ*, only contains a 1.5kbp regulatory sequence from the 5' region of the *dpp* gene [59]. We found that Bab1 and Bab2 are necessary for expression of this reporter in niche cells at the prepupal stage, but Bab2 is not sufficient for ectopic expression of *dppP4-lacZ* in adult germaria. In contrast, the second *dpp* transgene, containing a much larger genomic region including the *dpp* coding region and surrounding sequences (about 44kbp, [58]), was sensitive both to reduced levels of Bab proteins in larval and adult niches, and to overexpression of Bab2 in outside of niches in adult germaria. Bab proteins have been shown to bind A/T rich sequences with TA or TAA repeats [39] and the *dppP4-lacZ* 1.5kbp regulatory sequence, though containing A/T rich sequences which may bind Bab proteins, do not contain the consensus sequence (TAAATATAATTG), nor the *in vitro* determined optimal binding sequences of 3 or 4 TAA motifs in a row. The larger 44kbp genomic *dpp-nls-GFP* transgene, of course, contains many potential Bab protein-binding sites so it is probably not surprising that these two transgenes do not respond exactly the same to changes in Bab levels. Finally, a study in the embryo has shown enrichment of a Bab2:GFP fusion protein on two fragments of about 400bp in the 5' region of the *dpp* gene [65], and these do not overlap the *dppP4-lacZ* fragment which is nonetheless also in this region of the gene. Therefore, Bab proteins may regulate *dpp* expression directly.

### Complementary roles for Bab1/Bab2 and En/Inv in larval and adult GSC niches

In the developing ovary, *en/inv* are expressed specifically in niches during their formation. Our results show that their functions, however, are not essential for niche formation. Indeed, we found that when En/Inv were depleted efficiently by RNAi in developing niche cells during larval stages, the same number of correctly formed TFs, and TF cells and CCs per TF were found as in controls. This is consistent with the observation of larval ovaries upon En/Inv depletion in Allbee *et al*. [66], using a different niche cell Gal4 driver and the same *en/inv* RNAi transgenes used in the present study. In contrast, another study, based on induction of clones of ovarian somatic cells homozygous for a null allele for both *en/inv*, concluded that these genes are involved specifically for proper TF cell alignment within individual stacks in larval ovaries [36]. The difference with the results presented here may be related to the different approaches used to abolish *en/inv* expression. With the RNAi approach used here, all niche cells are depleted of En/Inv, while *en/inv* null mutant clone induction used in Bolívar *et al*. [36] led to the formation of stacks with both wild-type and mutant TF cells. Possible heterogeneity in the identity between the two populations of cells may have impeded normal interaction between TF cells during the intercalation process leading to TF formation. We cannot exclude that the RNAi approach, though leading to undetectable levels of En/Inv proteins, may nonetheless not have depleted En/Inv proteins completely. However, the *babG* driver used for *en/inv* RNAi induction is expressed early in L3 stage, before niche formation, and is strongly and homogeneously expressed in all niche cells, contrary to *hhG* which, nevertheless, gave a very strong TF formation defect with *bab1/bab2* RNAi transgenes.

Our results also indicate that En/Inv are not essential for initial GSC establishment. In fact, when their levels were efficiently reduced, the proportion of prepupal niches associated with at least one GSC was the same as in the control, but the overall number of GSCs per ovary was about 30% lower than in the control at this stage. We cannot exclude that RNAi knockdown of *en/inv* in niches was not sufficient to produce a stronger decrease in initial GSC numbers. However, the fact that we were able to show that all of the GSCs present at the prepupal stage in this *en/inv* RNAi context were subsequently completely lost between pupal stages and the first day after eclosion suggests a defect in GSC maintenance rather than in GSC establishment. In addition, when expression of *en/inv* was restored in niche cells from the beginning of pupariation, at least one GSC was present in more than 60% of adult germaria. These results suggest that the known function of En/Inv for GSC maintenance in the adult [17,23], may be necessary for this process even earlier, as soon as functional niches are formed. In contrast, upon strong reduction of Bab proteins in larval niche cells, the number of GSCs was about 90% lower than in the control at the prepupal stage, indicating a crucial role for Bab in initial GSC establishment.

In the adult ovary, a role for En/Inv in GSC maintenance through direct regulation of *dpp* expression has been shown [17], but this had not been tested for Bab proteins until now. Using the temperature-sensitive Gal80^TS^/UAS/Gal4 system to knockdown *bab1* and *bab2* only after GSC establishment at the pupal stage, we found that ovaries of 7-day old females present a significantly lower mean number of GSCs per germarium than control ovaries, as well as germaria with no GSCs and even no GCs at all, none of which was found in the control. This could not be attributed to a loss of *en/inv* expression in these cells. There was however, a slight but significant, lower mean number of CCs per niche when compared to the control (6.2 and 6.8, respectively). Nonetheless, the almost undetectable expression of the *dpp-nlsGFP* transgene in adult CCs strongly depleted of Bab proteins indicates that the loss of GSCs is most likely due to loss of *dpp* expression. The adult GSC loss phenotype is however much stronger when En/Inv proteins are depleted (this study and [17,23]). These results indicate that Bab1 and Bab2 contribute to GSC maintenance in adult ovaries likely via control of *dpp* expression in CCs.

Therefore, we propose that Bab proteins are the major players for initial GSC establishment through control of *dpp* expression in CCs during larval stages, and that, as of this point, GSC maintenance is mainly supported by En/Inv. Bab function for initial GSC establishment may be due solely to the direct regulation of *dpp* expression levels by these proteins, as shown for En for adult GSC maintenance, or to indirect regulation through specific targets of other transcription factors. Indeed, *Lmx1a*, encoding a LIM-homeodomain transcription factor expressed in somatic apical cells, TF cells and CCs in the prepupal ovary, is necessary for ovary morphogenesis similar to Bab proteins [66]. In addition, Bab proteins were shown to be necessary for *Lmx1a* expression. Thus, Lmx1a is a Bab target, either direct or indirect, and should be tested in the future for mediation of the role of Bab on *dpp* expression.

### Bab proteins are involved in homeostasis of GSCs in adult ovaries

We have shown that during ovary development, Bab proteins are necessary in niches for BMP pathway activation in initial GSCs, while they also contribute to GSC maintenance in the adult ovary. In both cases, this is associated with Bab positive control of expression of transgenes containing *dpp* transcriptional regulatory sequences, and independent of En/Inv. We also found that Bab2 overexpression in ECs and prefollicle cells in adult germaria was sufficient to produce excess GSCs with activated BMP/Dpp pathway, leading to the formation of tumorous germaria. This GSC-like tumorous phenotype was similar to that previously described for germarial overexpression of *dpp* [8,18] and *en* [17,24], and also upon JAK/STAT signaling ectopic activation [19,21]. Importantly, the GSC-like tumorous phenotype observed in germaria overexpressing *bab2* was independent of En/Inv. Our results indicate that a *dpp* genomic transgene is expressed in a significantly greater number of somatic germarial cells upon *bab2* overexpression than in the control and some of these are positioned in the region between the niche and prefollicle cells where *dpp* and this transgene are not expressed normally. This study thus identifies a new role for Bab proteins in GSC niches in regulating GSC establishment and homeostasis through activation of the BMP/Dpp pathway, thereby adding another level of complexity to be integrated into this system.

## Materials and methods

### Fly stocks

We used *hedgehog-Gal4* (gift from P. Therond and hereafter called *hhG*), a Gal4-expressing enhancer trap insertion in the *hh* locus, to target niche cells during their differentiation, and *bab*^*PGal4-2*^ (gift from J.L. Couderc and hereafter called *babG*), a Gal4-expressing enhancer trap insertion in the *bab l*ocus, to drive expression in all somatic cells of the larval ovary ([51] and present study). These drivers were combined with *UAS-dicer2* and *tub-Gal80*^*TS*^ (gift from J. Montagne) when indicated. Two different *UAS-GFP* transgenes (one associated with a Nuclear Localization Signal sequence, Bloomington Drosophila Stock Center, BDSC 4476, and one without, gift from A. Boivin), were used in combination with the *hhG* driver to mark niche cells. *The* RNAi lines *UAS-bab1*^*IR*^ (Vienna Drosophila Stock Center, VDRC 50285) and *UAS-bab2*^*IR*^ (VDRC 49042), and the *bab1* strong hypomorphic allele *bab*^*A128*^ [52] (gift from D.Godt) were used for the *bab* loss-of-function analysis. *bab*^*AR07*^,*FRT80B/TM6B* (gift from M. Boube) and *hs-FLP;; ubi-GFP*, *FRT80B* (gift from J. Montagne) lines were used to generate *bab* null mitotic cell clones. *bab*^*AR07*^ is a deletion mutation inactivating both *bab1* and *bab2* [34]. *UAS-en*^*IR*^ (VDRC 35697) and *UAS-inv*^*IR*^ (BDSC 41675) were used for the *engrailed/invected* knockdown analysis. The enhancer reporter line *P{PZ}1444* (*P1444-lacZ*) [67] and *bamP-GFP*, a GFP transcriptional reporter for *bam* expression ([20], Kyoto DGRC 118177) were used as cell-type specific markers. *E(spl)mβ-CD2*, in which the sequence encoding rat CD2 protein is inserted downstream of the *Enhancer of Split [E(spl)mβ]* promoter that is activated by Notch signaling, was used as a readout of Notch pathway activity [57] (gift from A. Bardin). *dpp-nlsGFP* (VDRC 318414) [58] and *P4-lacZ* [59] (gift from R. Xi) were used as *dpp* reporters. *dpp-nlsGFP* contains 43766 bp of the *dpp* genomic locus. *P4-lacZ* contains 1494bp of a *dpp* enhancer region. To drive ectopic and over-expression of *bab1* and *bab2*, we used *C587-Gal4* [32] (gift from T. Xie), *UAS-bab1* (BDSC, 6939) and *UAS-bab2*^*4-66*^ [61] (gift from A. Kopp).

### Experimental conditions

For all the experiments, flies were raised on standard cornmeal medium under uncrowded conditions. Prepupal ovaries correspond to ovaries extracted from 0–2 hour old white and slightly older (<3 hours) yellow prepupae.

For pupal analysis of the effect of *bab* and *engrailed/invected* (*en/inv*) depletion in niche cells with *bab*^*A128*^, *hhG*, *babG*, and RNAi lines, crosses were started at 25°C, parents removed 24-to-48h later, descendants were then transferred to 29°C and ovaries from prepupae were dissected.

To generate *bab* mosaic mutant prepupal ovaries, *bab*^*AR07*^, *FRT80B/TM6B* flies were crossed to *hs-FLP;; ubi-GFP*, *FRT80B* flies at 25°C. Clones marked by absence of GFP were induced by one 1-hour heat shock at 38°C at the end of the second instar larval stage, and prepupae were dissected for ovary analysis 50h after heat shock.

Transient expression of the *UAS-engrailed*^*IR*^ and *UAS-invected*^*IR*^ transgenes was achieved using *tub-Gal80*^*TS*^ *and babG*. For analysis of adult (1-day old) ovaries upon RNAi-mediated knockdown of *engrailed/invected* (*en/inv*), two conditions were used: 18°C (from crosses to dissection) for the control and 29°C (crosses at 25°C and transferred 24-to-48h later to 29°C) for RNAi activation. To test the effect of the depletion only during niche formation, *en/inv* were reexpressed at the prepupal stage by shifting prepupae from 29°C to 18°C for the rest of development.

Pupal and adult knockdown of *bab1* and *bab2* in *G80*^*TS*^*; hhG/UAS-bab1*^*IR*^, *UAS-bab2*^*IR*^ flies for comparison with in *G80*^*TS*^*; hhG/+* control flies was achieved raising females at 18°C to the young pupal stage (24h after puparium formation, APF), shifted to 29°C until eclosion and then shifted to 31°C for 7 days.

To analyze the effect on prepupal ovaries of *bab1* ectopic expression or *bab2* overexpression in somatic cells of larval ovaries, crosses were conducted at 25°C for 24-to-48h and descendants were then transferred either to 29°C or maintained at 25°C until dissection of prepupal ovaries. For *bab1* and *bab2* gain-of-function experiments in the adult, individuals were raised at 18°C and then shifted to 29°C or to 31°C just after eclosion of females as indicated in the figures legends. Ovaries from 10 day-old females were dissected.

### Immunostaining

Prepupal ovaries were dissected in PBS medium, fixed in 4% formaldehyde (R1026, Agar Scientific) for 20 to 30 minutes, and washed 3 x 10 minutes in PBT (PBS supplemented with 0.3% tritonX-100 –Sigma T8787). Ovaries were then incubated in a blocking solution (PBTA: PBT supplemented with 1% BSA–Sigma A3059) for a minimum of 20 minutes. Primary antibodies were diluted in PBTA and ovaries were incubated in this solution for 6 hours at room temperature or overnight at 4°C. The following primary antibodies were used: rabbit anti-Smad3 (1:200, ab52903, Abcam); rabbit anti-Bab1 (1:4000, gift from T. Williams); rabbit anti-GFP (1:200, FP-37151B, Interchim); rat anti-Bab2 (1:4000, gift from J-L. Couderc); rat IgM anti-Vasa (1:500, Developmental Studies Hybridoma Bank-DSHB); mouse anti-En/Inv recognizing both paralogs (1:200, 4D9, DSHB); guinea-pig anti GFP (1:200, 132 002, Synaptic System); mouse anti-β-Gal (1:400, 40-1a, DSHB); guinea pig anti-Traffic Jam (1:5000, gift from D. Godt); mouse anti-Delta (1:200, C594.9B, DSHB), mouse anti-rat CD2 (1:100, MCA154GA, Biorad); mouse anti Hts (1:200, DSHB). After primary antibody incubation, tissues were washed in PBT 3x10min, and incubated in PBTA for at least 30 minutes before incubation in secondary antibodies in PBTA. The following secondary antibodies were used: donkey anti-mouse Cy3 (715-165-151- Jackson Laboratories); and the following ones from Thermo Fisher Scientific, chicken anti-rabbit 488 (A21441); goat anti-rabbit 568 (A11011); goat anti-guinea pig 488 (A11073), anti-mouse 488 (A11029); anti-rat IgM 647 (A21248); anti-rat IgG 647 (A21247). Nuclei were detected with DAPI (1:200, 1mg/ml) and F-actin with 647nm-fluorescent dye conjugated phalloidin (1:200, 65906, Sigma). After secondary antibody incubation, tissues were washed 3x10 min in PBT, and mounted between slide and coverslip in DABCO (D27602, Sigma) with 70% glycerol. For larval ovaries, a spacer was positioned between the slide and the coverslip to avoid ovary squashing. The lateral side of the prepupal ovary was identified by its contact with the fat body.

### Fluorescence microscopy, image analysis and statistics

Images were acquired with a 40x or 63x objective on a confocal laser-scanning microscope Leica TCS SP8 equipped with 405nm, 488nm, 552nm, and 638nm emission diodes. Same settings were used between the different genotypes in each experiment. Prepupal ovaries were acquired with 1μm steps between each section and AOTF/EOF compensation was used to increase diode percentage during acquisition using the LasX software. Adult ovary images were acquired with 1μm steps between each section.

Images represent either a projection of 2–4 consecutive sections, full tissue stack, or a 3D reconstruction of the entire tissue. Images were processed with Fiji [68].

Counting TF number in prepupal ovaries was achieved through immunodetection of niche cell markers, such as GFP (*hhG+* cells), Bab1 or En/Inv, or DAPI labeling of flattened TF cell nuclei. Both lateral views and 3D reconstructions from the anterior pole of ovaries were used for this quantification. To distinguish TF cells from CCs within niches, labeling of F-actin, which delimits cell contours and/or DAPI labeling of TF cell nuclei was used. Niche cells with contours covering the entire width of the TF and with flattened nuclei were considered as TF cells, and more basal cells contacting GCs that did not cover the width of the TF and had round nuclei, as CCs (See Fig 1B, compare green and yellow brackets, respectively).

*hhG+* cell flattening of control prepupal ovaries (*hhG>GFP*) or upon depletion of Bab proteins (*hhG>GFP*, *bab1*^*IR*^, *bab2*^*IR*^) was quantified with GFP immunostaining marking *hhG+* cells and fluorescent phalloidin-bound F-actin marking cell contours in projections of 5 consecutive sections separated by 1μm. The width and height between cell contours of individual *hhG+* cells were measured, and the degree of flattening corresponded to the ratio between the width and height.

For comparison of En/Inv levels between control niche cells and niche cells depleted of Bab proteins (*bab*^*AR07*^ null allele and RNAi-mediated depletion), the confocal section presenting the biggest area for individual nuclei was used to quantify fluorescence intensity of En/Inv levels using the RawIntDen tool in Fiji.

To quantify GSC number in both prepupal ovaries and adult niches, we used nuclear pMad staining in GCs as a readout of Dpp pathway activity. pMad staining levels in nuclei were very variable between GCs of same ovary and between ovaries of a same experiment ranging from very high to very low when detected with Royal LUT. GCs presenting relatively low but unambiguous nuclear pMad staining were considered separately from GCs with high pMad+ GCs but both were considered as GSCs since pMad detection reflects high or moderate activity of Dpp/BMP pathway (see Fig 1D’ and 11D”).

Prism 7 (GraphPad Software) was used for statistical tests and for the generation of graphs. Values are presented as means + s.d., p-value calculated using a two-tailed Student's t-test or Mann Whitney test when two conditions were compared, or an ANOVA one-way test or Kruskal-Wallis test for comparisons between multiple conditions. Results are indicated with: NS (Not Significant—p>0.05); * (0.05>p>0.01); ** (0.01>p>0.001); *** (0.001>p>0.0001) and **** (p<0.0001). The data used to make all the figures and statistical analyses in this study can be found in S1 Dataset.

## Supporting information

S1 Fig *bab1 and bab2* expression patterns in developing niches and Bab1 and Bab2 depletion using UAS-RNAi transgenes and the *hedgehog(hh)-Gal4* driver. Whole mount immunostaining of L2 (A-A’, E, I-I”, K-K”), early L3 (EL3, B-B’, F), mid-L3 (ML3, C-C’, G, J-J”, L-L”) and late L3 (LL3, D-D’, H) ovaries. Anterior is up. Scale bar: 10 μm. Precursor Terminal Filament (TF) cells and TFs are encircled by blue dotted lines in all images except E-H. (A-H) Control larval ovaries expressing a *UAS-GFP* construct with a *hedgehog(hh)-Gal4 driver (hhG>GFP*). Experimental ovaries expressing RNAi directed against *bab2 (hhG>GFP*, *bab2*^*IR*^) in the absence (I-J”) or presence (K-L”) of a homozygous *bab1* strong hypomorphic mutation (*bab*^*A128*^*; hhG>GFP*, *bab2*^*IR*^). (A, B, I, K) *hhG>GFP* is expressed in a few anterior somatic cells of L2 and early L3 ovaries whose position in the ovary might correspond to precursor TF cells. (C) In the mid-L3 ovary, *hhG+* cells are more numerous and have intercalated leading to formation of short TF cell stacks, while full sized TFs are completely formed by late the L3 stage (D). Bab1 is not detectable during the L2 and early L3 stages (A’, B’), but accumulates during mid- and late-L3 stages in *hhG+* cells that form TFs (C',D'). (E-H) Bab2 is expressed in all somatic cells of L2 and L3 ovaries. (I-J”) In *hhG>GFP*, *bab2*^*IR*^ ovaries, Bab2 depletion can be observed in most *hhG+* cells from L2 and mid-late 3 stages (I-I”,J-J”, orange arrowheads), without a noticeable change in Bab1 levels (I’-J’). However, a few *hhG+* cells are not knocked down for *bab2* in *hhG>GFP*, *bab2*^*IR*^ (J, J”, blue arrowheads). (K-L”) In *bab*^*A128*^, *hhG>GFP*, *bab2*^*IR*^ ovaries, a variable level of Bab2 depletion is also observed in L2 and mid-L3 ovaries, (K,K",L,L”, orange and blue arrowheads). These ovaries do not present detectable Bab1 protein at the L2 (K') or at the mid-L3 (L’) stage due to the effect of the *bab*^*A128*^ allele (L').
(TIF)

S2 Fig Expression pattern of the *bab-Gal4* driver during L3 stages. (A-E) Ovaries of *bab-Gal4>UAS-GFP* L3 larvae raised at 25°C, immunostained for detection of GFP (green), and labeled for F-Actin with phalloidin (grey) to mark cell perimeters. Anterior is up. Scale bars: 10μm. *babG* is expressed in all somatic cells throughout L3 stages as visualized by *UAS-GFP* expression.
(TIF)

S3 Fig Bab2 is necessary for ovary development during larval stages. (A-B) Prepupal ovaries from individuals raised at 25°C during embryogenesis and the L1 stage, then shifted to 29°C. Prepupal ovaries were immunostained for detection of Engrailed/Invected (En/Inv, red) and labeled for F-Actin to mark cell perimeters. Anterior is up. Scale bars: 10μm. (A) In the control *G80*^*TS*^, *babG>+*, En/Inv are detected in TF cells and CCs. (B) Ovaries of *G80*^*TS*^, *babG>bab2*^*IR*^ prepupae are much smaller and more spherical than control ovaries. Very few En/Inv+ cells are present and TFs are not formed indicating severe morphogenesis and growth defects, including absence of GSC niche formation.
(TIF)

S4 Fig Almost complete reduction of Bab1 and partial reduction of Bab2 levels using shmiRNAs impedes TF formation and GSC establishment. (A-E) Prepupal ovaries immunostained for detection of GFP (green). Nuclei are labeled with DAPI (grey). Anterior is up. Scale bars: 10 μm. (A’,B’,C’,D’,E’) Higher magnifications of the niche regions of the corresponding ovaries. Yellow dotted lines encircle medial niche regions and blue dotted lines encircle lateral TFs. (A-C) Ovaries immunostained for Bab1 and Bab2. (A-A’) Control ovary expressing a *UAS-GFP* transgene under the control of a *hedgehog(hh)-Gal4* driver (*hhG>GFP*) and showing Bab1 (red) and Bab2 (yellow) levels in nuclei of cells in the niche region. (B-B’) Simultaneous RNAi for *bab1* and *bab2* was conducted by using the UAS-Gal4 system for expressing a ‘chained’ *bab1* and *bab2* shmiR transgene (*hhG>GFP*, *bab1-bab2*^*shmiR*^; Id#3–12) constructed by Roeske and co-workers (eLife. 2018;7:e32273). Bab1 levels are only somewhat lower than in the control, while Bab2 levels are clearly lower than in the control (B'). (C-C’, E-E’) Prepupal ovary expressing shmiRNAs directed against *bab1* and *bab2* in a strong hypomorphic *bab1* background (*bab*^*A128*^, *hhG>GFP*, *bab1-bab2*^*shmiR*^). Bab1 is undetectable and Bab2 levels are low (C’). *hhG*+ cells (green) have round nuclei and fail to form TFs (C’, yellow dotted lines). (D-E’) Prepupal ovaries immunostained for pMad (red) and Vasa (blue). In a control *hhG>GFP* ovary, pMad is detected in Germline Stem Cells (GSCs) (D’, arrows). In the medial part of a *bab*^*A128*^, *hhG>GFP*, *bab1-bab2*^*shmiR*^ ovary where Bab proteins are depleted (yellow dotted line, E’), GCs are present in immediate proximity to *hhG+* cells, but almost none of these GCs are GSCs since they are not pMad+ (arrowheads). In the lateral region, where normal TFs are formed (blue dotted line), pMad+ GSCs (arrow) are present in the niche. These results confirm those obtained using different genetic tools to reduce Bab1 and Bab2 levels in GSC niches (Fig 2 and Fig 3).
(TIF)

S5 Fig High levels of E-Cadherin are found between Germ Cells and niche cells depleted of Bab1 and Bab2. (A-C) Whole mount immunostaining of a *hhG>GFP*,*bab1IR*,*bab2IR* prepupal ovary. Anterior is up, medial is left. Scale bars: 10 μm. The dotted box encloses the lateral niches of the ovary. *bab1* and *bab2* were targeted by UAS-RNAi transgenes under the control of the *hhG* driver. *UAS-GFP* was used to visualize the cells in which the driver is active. (A, B) In the lateral region, where we have shown that Bab protein depletion is ineffective (correlated with low *hhG>GFP* expression, green), normal Terminal Filaments (TFs) are formed (Dapi, grey and *hhG>GFP*, green) and these are associated with GSCs presenting pMad (red, yellow arrows). In the medial part of the ovary where we have shown that Bab protein depletion is effective (correlated with high *hhG>GFP* expression, green), *hhG+* cells do not form TFs, and Germ Cells (GCs, red asterisks) very closely juxtaposed to *hhG+* cells do not express pMad, indicating they are not GSCs. (C) E-cadherin (E-Cadh, grey) is nonetheless present between these GCs and Bab depleted *hhG+* cells (orange arrowheads), as is the case between wild type lateral niches and adjacent GSCs (blue arrowheads). Therefore, the fact that GCs do not acquire GSC status when niche cells are depleted of Bab proteins is not likely due to diminished adhesion with the niche because of a problem in the level of E-cadherin-based adherens junctions between niche cells and GSCs.
(TIF)

S6 Fig Expression pattern of the *dpp-nlsGFP* transgene construct in adult germaria. (A) Adult germarium from a *dpp-nlsGFP* female raised at 25°C and immunostained for detection of GFP (green), Engrailed/Invected (En/Inv, red) and Traffic Jam(Tj)/Vasa (magenta). Nuclei are labeled with DAPI (grey). Anterior is up. Scale bars: 10μm. (A) Entire germarium and (A’-A”’) higher magnifications of the region framed with dotted lines in (A). GFP is present in Cap Cells co-marked by nuclear En/Inv and nuclear Tj (yellow brackets), and in some Escort Cells (blue brackets), but absent from Terminal Filament cells marked by En/Inv (green brackets). Expression of GFP was also detected in prefollicle cells (orange brackets).
(TIF)

S7 Fig Reduction of Bab proteins in niche cells only from pupal to adult stages leads to loss of GSCs. (A-B) Adult germaria from control females carrying transgenes for temperature-controlled GFP expression (*G80*^*TS*^*; hhG>UAS-GFP*). Anterior is left. Scale bars: 10μm (A) Females were raised at 18°C throughout development and shifted to 31°C upon eclosion for 7 days thereafter or (B) raised at 18°C until the early pupal stage, then shifted to 29°C until eclosion and finally transferred to 31°C for 7 days. Ovaries were immunostained for detection of GFP (green), Engrailed/Invected (En/inv, red) and Bab2 (cyan). CCs are marked by the presence of En and Bab2. (A’-A”,B’-B”) Higher magnifications of the corresponding niche regions in (A, B). (A’-A”) Shifting of *G80*^*TS*^, *hhG>GFP* adults to 31°C upon eclosion led to a mean of only 1.2 (s.d. = 1.4) CCs expressing GFP per germarium for a mean of 6.3 (s.d. = 1.2) total CCs per germarium (therefore only 19.5% of CCs per germarium expressed GFP, n = 40 germaria), showing that the *hhG* driver was not efficiently expressed in CCs at the adult stage under these conditions (S1 Dataset). (B-B”) Conversely, shifting adults of the same genotype at 24h after pupariation led to a mean of 5.8 (s.d. = 1.4) CCs expressing GFP per germarium for a mean of 5.9 (s.d. = 1.2) total CCs per germarium (indicating that 97.9% of CCs per germarium expressed GFP, n = 19), thereby showing normal adult expression of the *hhG* driver in CCs (S1 Dataset). (C-F) Adult germaria from control *G80*^*TS*^, *G80>GFP* (C-C”) and *bab*^*A128*^, *Gal80*^*TS*^, *hhG>bab2*^*IR*^ (D-F”) females raised under conditions allowing efficient expression of the *hhG* driver in adults but not in larvae during ovary development. Anterior is to the left. Scale bars: 10μm. Ovaries were immunostained for detection of En/inv (red), pMad (green) and Vasa (magenta). Bab depletion led mostly to rudimentary ovaries. Indeed, the number of ovaries containing ovarioles recovered was extremely low, since out of 10 ovaries, only 27 germaria were clearly identified, compared with the 180–200 germaria expected (considering 18–20 ovarioles/ovary). Among these, 44.4% contained one or two pMad+ GSCs (D’-D”), 22.2% contained pMad- GCs (E’-E”) and 33.3% were completely devoid of GCs (F-F”). Nuclei are labeled with DAPI (grey). (G-J”) Control (*G80*^*TS*^, *hhG>GFP*) and *bab*^*A128*^,*Gal80*^*TS*^,*hhG>bab2*^*IR*^ prepupal ovaries from females raised at 18°C immunostained for detection of En/Inv (red, G, H-H’, I, J-J’), Bab1 (green, G’, I’), Bab2 (yellow, G”, I”), pMad (green, H”, J”) and Vasa (magenta, H”, J”). Anterior is up. Scale bars: 10μm. (G’-J”) Higher magnifications of the corresponding niche regions of G-J. The level of Bab2 in *bab*^*A128*^, *G80*^*TS*^, *hhG>bab2*^*IR*^ prepupal ovaries from females raised at 18°C (I”) corresponds to that in control ovaries (G”), niches are correctly formed (I,J compare to G,H) and these niches contain pMad+ GSCs (J”).
(TIF)

S8 Fig Reduction of Bab2 in niche cells from the early pupal stage onwards does not affect GSC numbers in adult germaria. (A-B) Germaria from control *hhG>GFP* and *hhG>GFP*, *bab2*^*IR*^ adult females, raised at 18°C up to 24h after puparium formation to maintain the UAS/Gal4 system inactive, shifted to 29°C during the rest of pupal development and finally shifted to 31°C upon eclosion and for 7 days thereafter to activate *bab2*^*IR*^ only at the pupal and the adult stages. Ovaries were immunostained for detection of GFP (green), Bab1 (red) and Bab2 (yellow). Nuclei are labeled with DAPI (grey). (A’-A”’ to B’-B”’) Higher magnifications of the niche regions marked with dotted lines in A,B (Cap Cells, CCs are indicated by yellow brackets). Anterior is to the left. Scale bars: 10μm. In the control (A), both Bab1 and Bab2 are present in CCs. (B) In the presence of the *bab2*^*IR*^ transgene, Bab1 is present in CCs (B"), while Bab2 is undetectable (B‴) indicating efficient *bab2* knockdown. (C-D) Germaria from females of the same genotypes having undergone the same developmental temperature shifts as in (A,B) immunostained for GFP (green), pMad (red) and Vasa (cyan). Anterior is to the left. Scale bars: 10μm. For both genotypes, GSCs are present in the niche (C’-C” and D’-D”, yellow arrowheads). (E) Graph comparing the mean number of GSCs per germarium in adult control ovaries and ovaries in which Bab2 was efficiently reduced during pupal and adult stages. No statistical difference (NS) was observed between the two genetic contexts. Values are presented as means +s.d., p-values are calculated using a two-tailed t-test or a Mann-Whitney test. n: sample size.
(TIF)

S9 Fig The ectopic expression of *bab1* or the overexpression of *bab2* does not affect ovary morphogenesis. (A-C) Prepupal ovaries from females raised at 25°C, immunostained for Bab1 and Bab2 (Fiji Royal Lookup Table (LUT) indicating signal intensity, inset to the right) and Engrailed/Invected (En/Inv, red). Anterior is up. Scale bars: 10μm. (A) Control ovary (*C587G>+*) showing the accumulation of Bab1 (high in niche cells and low in Intermingled Cells (ICs, white brackets)). (B) The *C587G* driver coupled with *UAS-bab1* allows increased accumulation of Bab1 in some ICs (arrowheads). (C') The *C587G* driver coupled with *UAS-bab2* also leads to increased accumulation of Bab2 in some ICs (arrowheads). (B',C vs. A',A) No evidence of cross-regulation between Bab1 and Bab2 is observed. (B”,C”) Neither of these transgenes causes the presence of ectopic En/Inv in ICs with an excess of Bab1 or Bab2 (arrowheads) compared to the control (A"). The overall organization of these ovaries does not seem disturbed when compared to the control.
(TIF)

S10 Fig Adult *bab1* ectopic expression and *bab2* overexpression using the *C587-Gal4* driver. (A-C) Germaria from ovaries of 10-day old females immunostained for Bab1 (green) and Bab2 (red). DAPI nuclear labeling is in grey. Anterior is up. Scale bars: 10μm. (A-A") In the control (*C587G>+*), Bab1 (A’) and Bab2 (A”) are present in niche cells, mainly in the Cap Cells (CCs) (yellow bracket) and overlying Terminal Filaments (TFs). Bab2 can also be detected faintly in some Escort Cells (ECs) (A”, blue arrowhead) and more posterior somatic cells (A", yellow arrowhead). (B-B”) Ectopic expression of *bab1* (*C587G>UAS-bab1*, B’) or overexpression of *bab2* (*C587G>UAS-bab2*, C") causes elevated accumulation of the corresponding proteins in ECs and prefollicle cells (blue and yellow arrowheads, respectively). Cross regulation between *bab1* and *bab2* does not occur (B” and C’, blue and yellow arrowheads).
(TIF)

S1 Dataset Data used to make all the figures and statistical analyses. (XLSX)

S1 Text Supplemental materials and experimental procedures. (DOCX)
