# Supplementary figures and images for: The Bric-à-Brac BTB/POZ transcription factors are necessary in niche cells for germline stem cells establishment and homeostasis through control of BMP/DPP signaling in the Drosophila melanogaster ovary
Source: PLoS Genet. 2020 Nov 5;16(11):e1009128. doi: 10.1371/journal.pgen.1009128 (PMC7643948; doi:10.1371/journal.pgen.1009128)

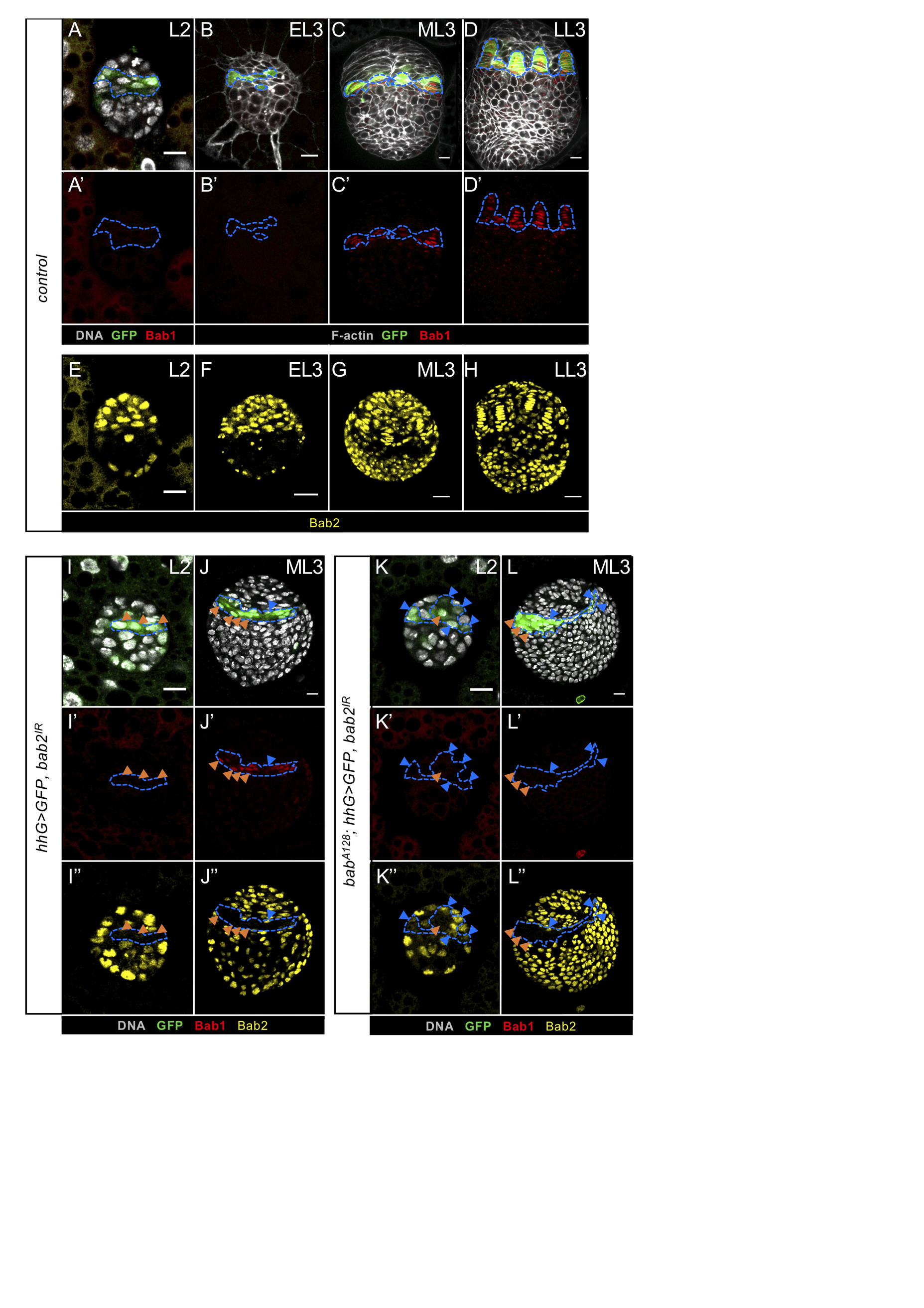

Supplement: S1 Fig — Whole mount immunostaining of L2 (A-A’, E, I-I”, K-K”), early L3 (EL3, B-B’, F), mid-L3 (ML3, C-C’, G, J-J”, L-L”) and late L3 (LL3, D-D’, H) ovaries. Anterior is up. Scale bar: 10 μm. Precursor Terminal Filament (TF) cells and TFs are encircled by blue dotted lines in all images except E-H. (A-H) Control larval ovaries expressing a UAS-GFP construct with a hedgehog(hh)-Gal4 driver (hhG>GFP). Experimental ovaries expressing RNAi directed against bab2 (hhG>GFP, bab2IR) in the absence (I-J”) or presence (K-L”) of a homozygous bab1 strong hypomorphic mutation (babA128; hhG>GFP, bab2IR). (A, B, I, K) hhG>GFP is expressed in a few anterior somatic cells of L2 and early L3 ovaries whose position in the ovary might correspond to precursor TF cells. (C) In the mid-L3 ovary, hhG+ cells are more numerous and have intercalated leading to formation of short TF cell stacks, while full sized TFs are completely formed by late the L3 stage (D). Bab1 is not detectable during the L2 and early L3 stages (A’, B’), but accumulates during mid- and late-L3 stages in hhG+ cells that form TFs (C',D'). (E-H) Bab2 is expressed in all somatic cells of L2 and L3 ovaries. (I-J”) In hhG>GFP, bab2IR ovaries, Bab2 depletion can be observed in most hhG+ cells from L2 and mid-late 3 stages (I-I”,J-J”, orange arrowheads), without a noticeable change in Bab1 levels (I’-J’). However, a few hhG+ cells are not knocked down for bab2 in hhG>GFP, bab2IR (J, J”, blue arrowheads). (K-L”) In babA128, hhG>GFP, bab2IR ovaries, a variable level of Bab2 depletion is also observed in L2 and mid-L3 ovaries, (K,K",L,L”, orange and blue arrowheads). These ovaries do not present detectable Bab1 protein at the L2 (K') or at the mid-L3 (L’) stage due to the effect of the babA128 allele (L'). (TIF) [file pgen.1009128.s001.tif]

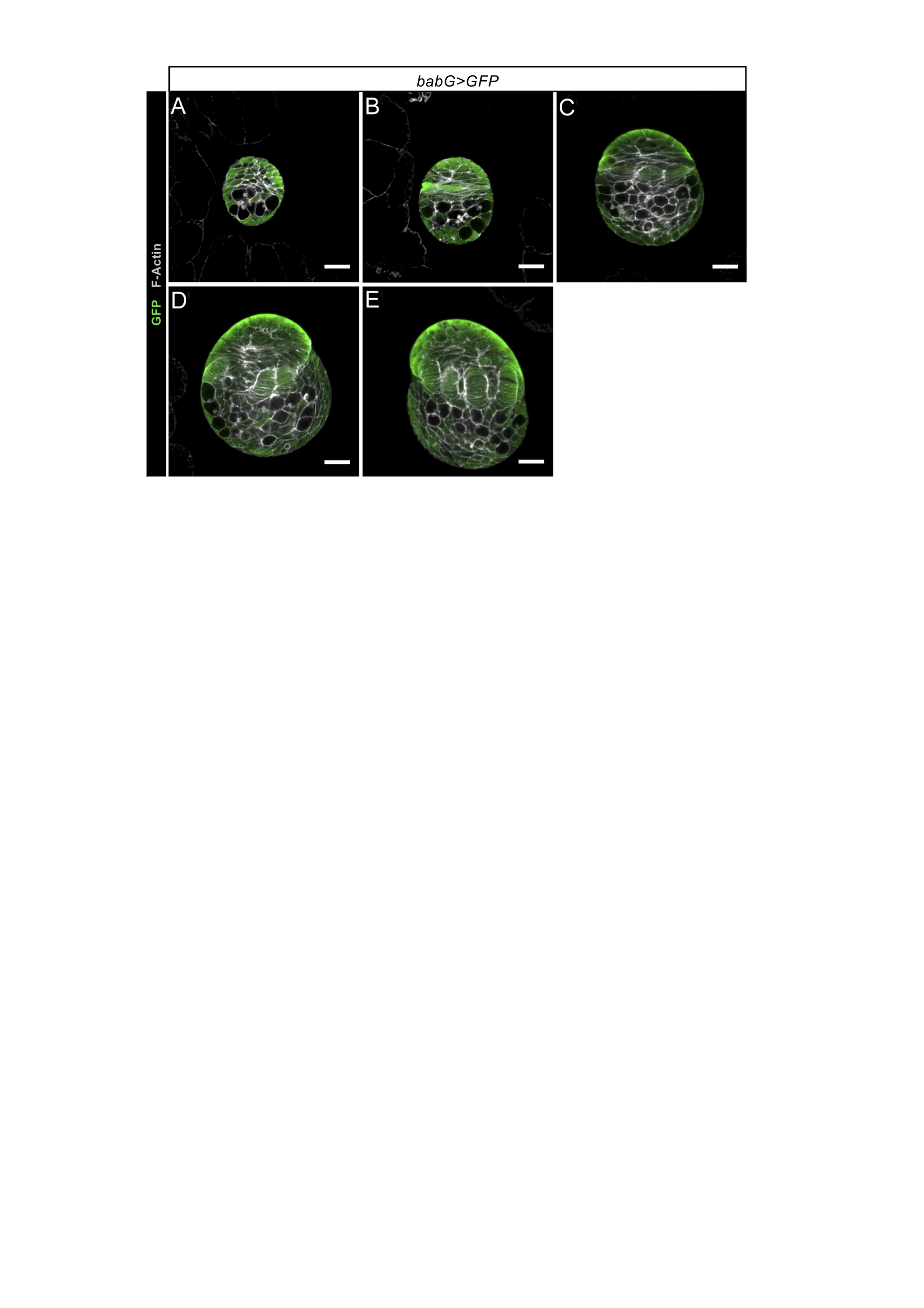

Supplement: S2 Fig — (A-E) Ovaries of bab-Gal4>UAS-GFP L3 larvae raised at 25°C, immunostained for detection of GFP (green), and labeled for F-Actin with phalloidin (grey) to mark cell perimeters. Anterior is up. Scale bars: 10μm. babG is expressed in all somatic cells throughout L3 stages as visualized by UAS-GFP expression. (TIF) [file pgen.1009128.s002.tif]

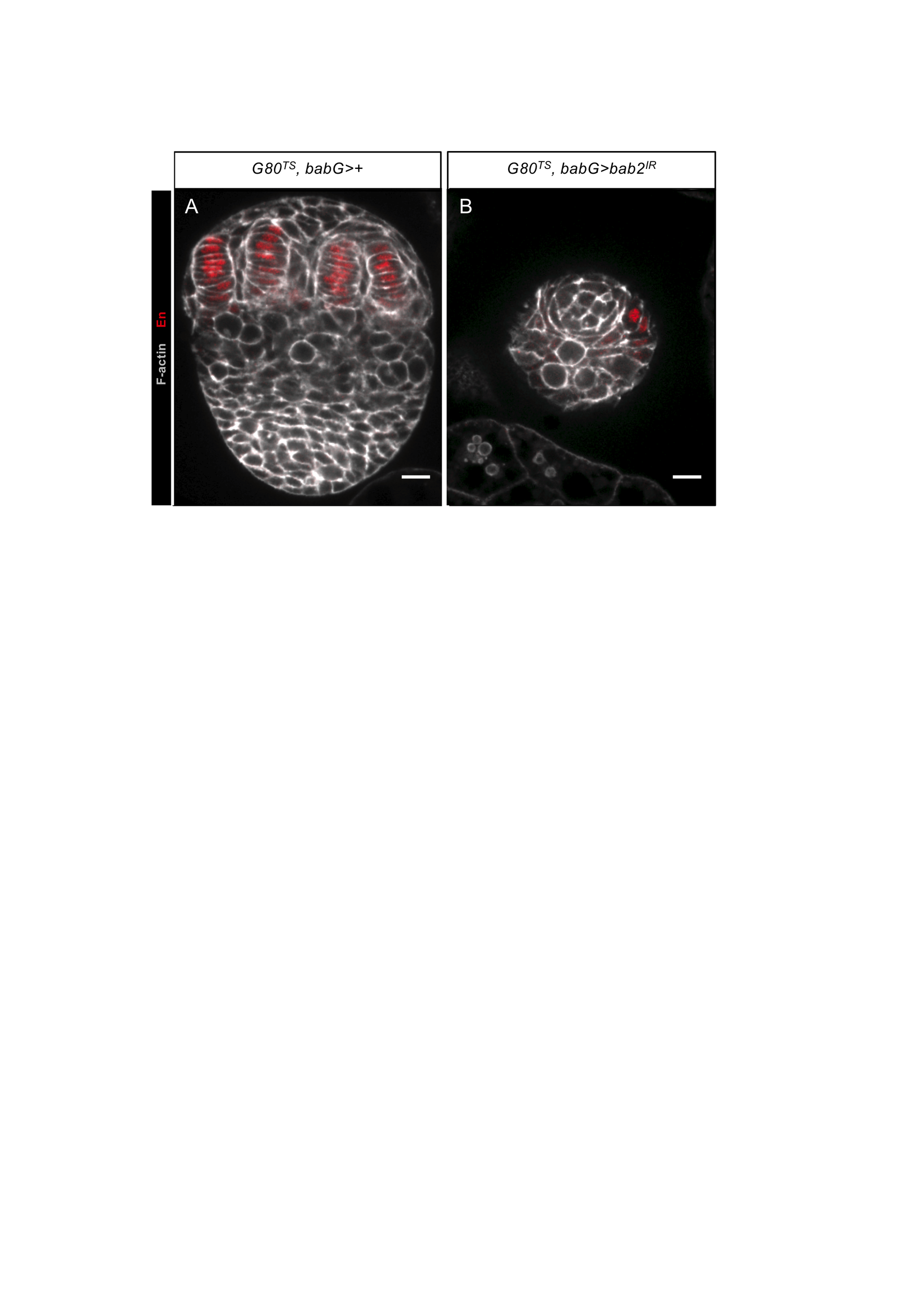

Supplement: S3 Fig — (A-B) Prepupal ovaries from individuals raised at 25°C during embryogenesis and the L1 stage, then shifted to 29°C. Prepupal ovaries were immunostained for detection of Engrailed/Invected (En/Inv, red) and labeled for F-Actin to mark cell perimeters. Anterior is up. Scale bars: 10μm. (A) In the control G80TS, babG>+, En/Inv are detected in TF cells and CCs. (B) Ovaries of G80TS, babG>bab2IR prepupae are much smaller and more spherical than control ovaries. Very few En/Inv+ cells are present and TFs are not formed indicating severe morphogenesis and growth defects, including absence of GSC niche formation. (TIF) [file pgen.1009128.s003.tif]

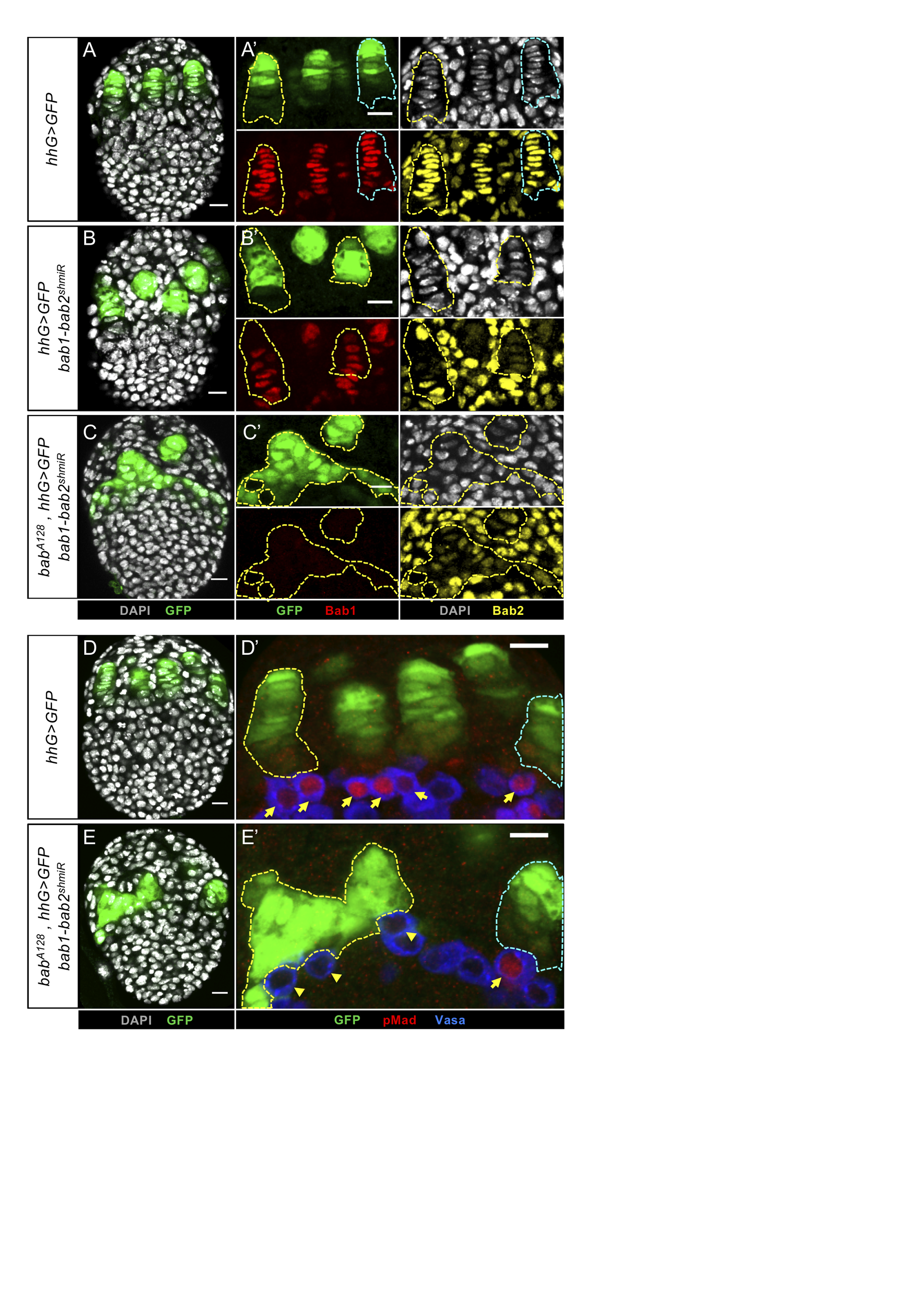

Supplement: S4 Fig — (A-E) Prepupal ovaries immunostained for detection of GFP (green). Nuclei are labeled with DAPI (grey). Anterior is up. Scale bars: 10 μm. (A’,B’,C’,D’,E’) Higher magnifications of the niche regions of the corresponding ovaries. Yellow dotted lines encircle medial niche regions and blue dotted lines encircle lateral TFs. (A-C) Ovaries immunostained for Bab1 and Bab2. (A-A’) Control ovary expressing a UAS-GFP transgene under the control of a hedgehog(hh)-Gal4 driver (hhG>GFP) and showing Bab1 (red) and Bab2 (yellow) levels in nuclei of cells in the niche region. (B-B’) Simultaneous RNAi for bab1 and bab2 was conducted by using the UAS-Gal4 system for expressing a ‘chained’ bab1 and bab2 shmiR transgene (hhG>GFP, bab1-bab2shmiR; Id#3–12) constructed by Roeske and co-workers (eLife. 2018;7:e32273). Bab1 levels are only somewhat lower than in the control, while Bab2 levels are clearly lower than in the control (B'). (C-C’, E-E’) Prepupal ovary expressing shmiRNAs directed against bab1 and bab2 in a strong hypomorphic bab1 background (babA128, hhG>GFP, bab1-bab2shmiR). Bab1 is undetectable and Bab2 levels are low (C’). hhG+ cells (green) have round nuclei and fail to form TFs (C’, yellow dotted lines). (D-E’) Prepupal ovaries immunostained for pMad (red) and Vasa (blue). In a control hhG>GFP ovary, pMad is detected in Germline Stem Cells (GSCs) (D’, arrows). In the medial part of a babA128, hhG>GFP, bab1-bab2shmiR ovary where Bab proteins are depleted (yellow dotted line, E’), GCs are present in immediate proximity to hhG+ cells, but almost none of these GCs are GSCs since they are not pMad+ (arrowheads). In the lateral region, where normal TFs are formed (blue dotted line), pMad+ GSCs (arrow) are present in the niche. These results confirm those obtained using different genetic tools to reduce Bab1 and Bab2 levels in GSC niches (Fig 2 and Fig 3). (TIF) [file pgen.1009128.s004.tif]

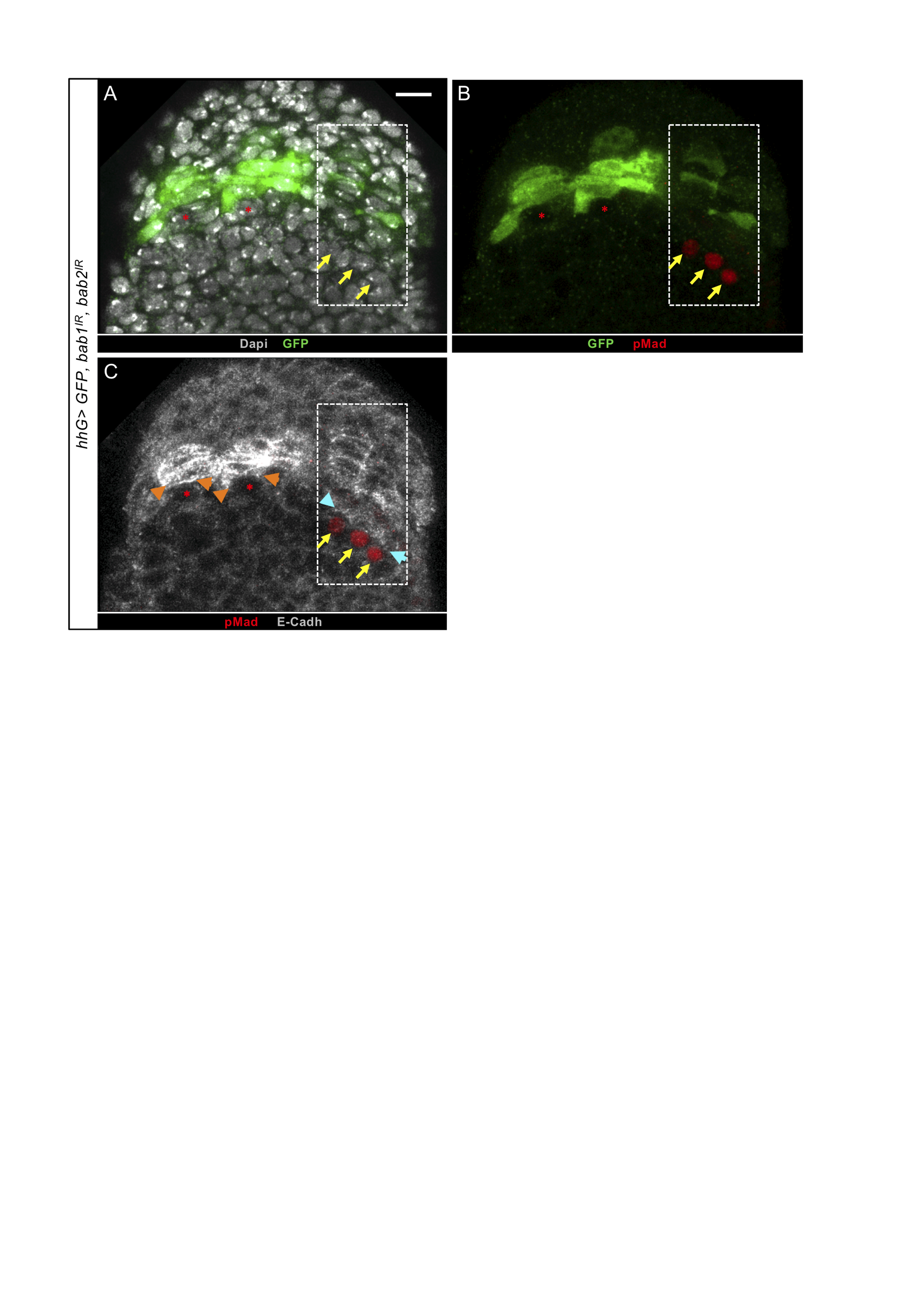

Supplement: S5 Fig — (A-C) Whole mount immunostaining of a hhG>GFP,bab1IR,bab2IR prepupal ovary. Anterior is up, medial is left. Scale bars: 10 μm. The dotted box encloses the lateral niches of the ovary. bab1 and bab2 were targeted by UAS-RNAi transgenes under the control of the hhG driver. UAS-GFP was used to visualize the cells in which the driver is active. (A, B) In the lateral region, where we have shown that Bab protein depletion is ineffective (correlated with low hhG>GFP expression, green), normal Terminal Filaments (TFs) are formed (Dapi, grey and hhG>GFP, green) and these are associated with GSCs presenting pMad (red, yellow arrows). In the medial part of the ovary where we have shown that Bab protein depletion is effective (correlated with high hhG>GFP expression, green), hhG+ cells do not form TFs, and Germ Cells (GCs, red asterisks) very closely juxtaposed to hhG+ cells do not express pMad, indicating they are not GSCs. (C) E-cadherin (E-Cadh, grey) is nonetheless present between these GCs and Bab depleted hhG+ cells (orange arrowheads), as is the case between wild type lateral niches and adjacent GSCs (blue arrowheads). Therefore, the fact that GCs do not acquire GSC status when niche cells are depleted of Bab proteins is not likely due to diminished adhesion with the niche because of a problem in the level of E-cadherin-based adherens junctions between niche cells and GSCs. (TIF) [file pgen.1009128.s005.tif]

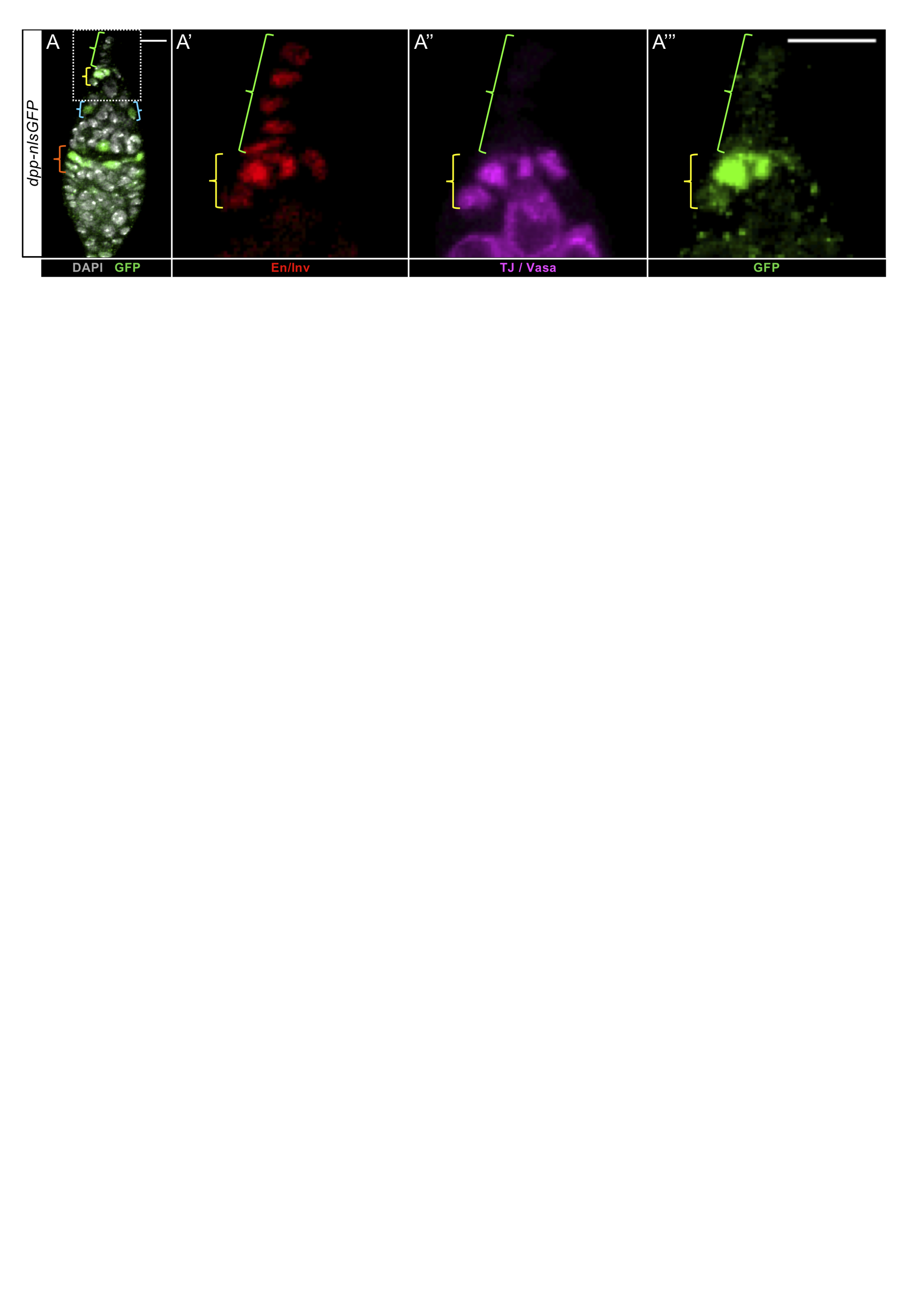

Supplement: S6 Fig — (A) Adult germarium from a dpp-nlsGFP female raised at 25°C and immunostained for detection of GFP (green), Engrailed/Invected (En/Inv, red) and Traffic Jam(Tj)/Vasa (magenta). Nuclei are labeled with DAPI (grey). Anterior is up. Scale bars: 10μm. (A) Entire germarium and (A’-A”’) higher magnifications of the region framed with dotted lines in (A). GFP is present in Cap Cells co-marked by nuclear En/Inv and nuclear Tj (yellow brackets), and in some Escort Cells (blue brackets), but absent from Terminal Filament cells marked by En/Inv (green brackets). Expression of GFP was also detected in prefollicle cells (orange brackets). (TIF) [file pgen.1009128.s006.tif]

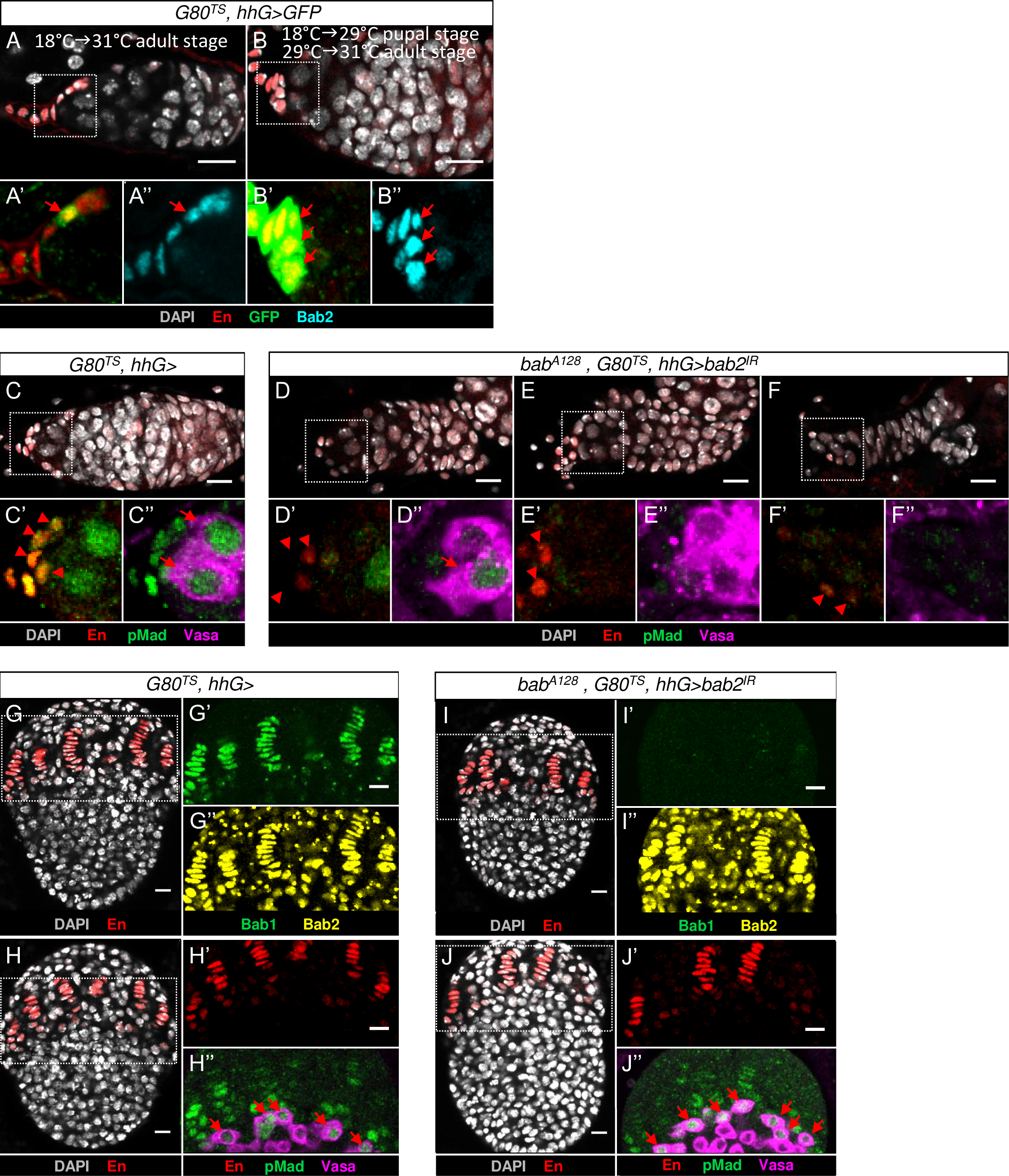

Supplement: S7 Fig — (A-B) Adult germaria from control females carrying transgenes for temperature-controlled GFP expression (G80TS; hhG>UAS-GFP). Anterior is left. Scale bars: 10μm (A) Females were raised at 18°C throughout development and shifted to 31°C upon eclosion for 7 days thereafter or (B) raised at 18°C until the early pupal stage, then shifted to 29°C until eclosion and finally transferred to 31°C for 7 days. Ovaries were immunostained for detection of GFP (green), Engrailed/Invected (En/inv, red) and Bab2 (cyan). CCs are marked by the presence of En and Bab2. (A’-A”,B’-B”) Higher magnifications of the corresponding niche regions in (A, B). (A’-A”) Shifting of G80TS, hhG>GFP adults to 31°C upon eclosion led to a mean of only 1.2 (s.d. = 1.4) CCs expressing GFP per germarium for a mean of 6.3 (s.d. = 1.2) total CCs per germarium (therefore only 19.5% of CCs per germarium expressed GFP, n = 40 germaria), showing that the hhG driver was not efficiently expressed in CCs at the adult stage under these conditions (S1 Dataset). (B-B”) Conversely, shifting adults of the same genotype at 24h after pupariation led to a mean of 5.8 (s.d. = 1.4) CCs expressing GFP per germarium for a mean of 5.9 (s.d. = 1.2) total CCs per germarium (indicating that 97.9% of CCs per germarium expressed GFP, n = 19), thereby showing normal adult expression of the hhG driver in CCs (S1 Dataset). (C-F) Adult germaria from control G80TS, G80>GFP (C-C”) and babA128, Gal80TS, hhG>bab2IR (D-F”) females raised under conditions allowing efficient expression of the hhG driver in adults but not in larvae during ovary development. Anterior is to the left. Scale bars: 10μm. Ovaries were immunostained for detection of En/inv (red), pMad (green) and Vasa (magenta). Bab depletion led mostly to rudimentary ovaries. Indeed, the number of ovaries containing ovarioles recovered was extremely low, since out of 10 ovaries, only 27 germaria were clearly identified, compared with the 180–200 germaria expected (considering 18–20 [file pgen.1009128.s007.tif]

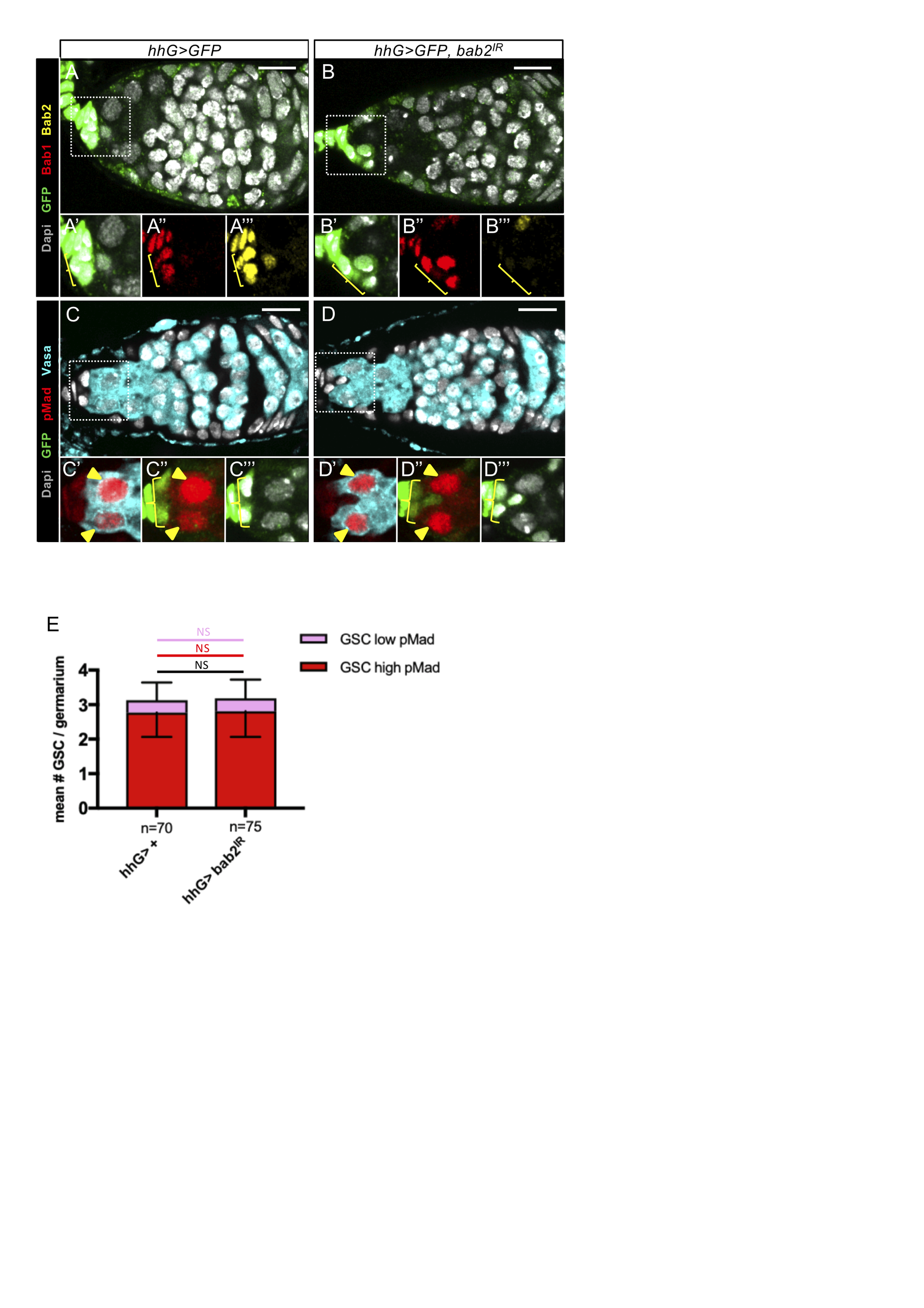

Supplement: S8 Fig — (A-B) Germaria from control hhG>GFP and hhG>GFP, bab2IR adult females, raised at 18°C up to 24h after puparium formation to maintain the UAS/Gal4 system inactive, shifted to 29°C during the rest of pupal development and finally shifted to 31°C upon eclosion and for 7 days thereafter to activate bab2IR only at the pupal and the adult stages. Ovaries were immunostained for detection of GFP (green), Bab1 (red) and Bab2 (yellow). Nuclei are labeled with DAPI (grey). (A’-A”’ to B’-B”’) Higher magnifications of the niche regions marked with dotted lines in A,B (Cap Cells, CCs are indicated by yellow brackets). Anterior is to the left. Scale bars: 10μm. In the control (A), both Bab1 and Bab2 are present in CCs. (B) In the presence of the bab2IR transgene, Bab1 is present in CCs (B"), while Bab2 is undetectable (B‴) indicating efficient bab2 knockdown. (C-D) Germaria from females of the same genotypes having undergone the same developmental temperature shifts as in (A,B) immunostained for GFP (green), pMad (red) and Vasa (cyan). Anterior is to the left. Scale bars: 10μm. For both genotypes, GSCs are present in the niche (C’-C” and D’-D”, yellow arrowheads). (E) Graph comparing the mean number of GSCs per germarium in adult control ovaries and ovaries in which Bab2 was efficiently reduced during pupal and adult stages. No statistical difference (NS) was observed between the two genetic contexts. Values are presented as means +s.d., p-values are calculated using a two-tailed t-test or a Mann-Whitney test. n: sample size. (TIF) [file pgen.1009128.s008.tif]

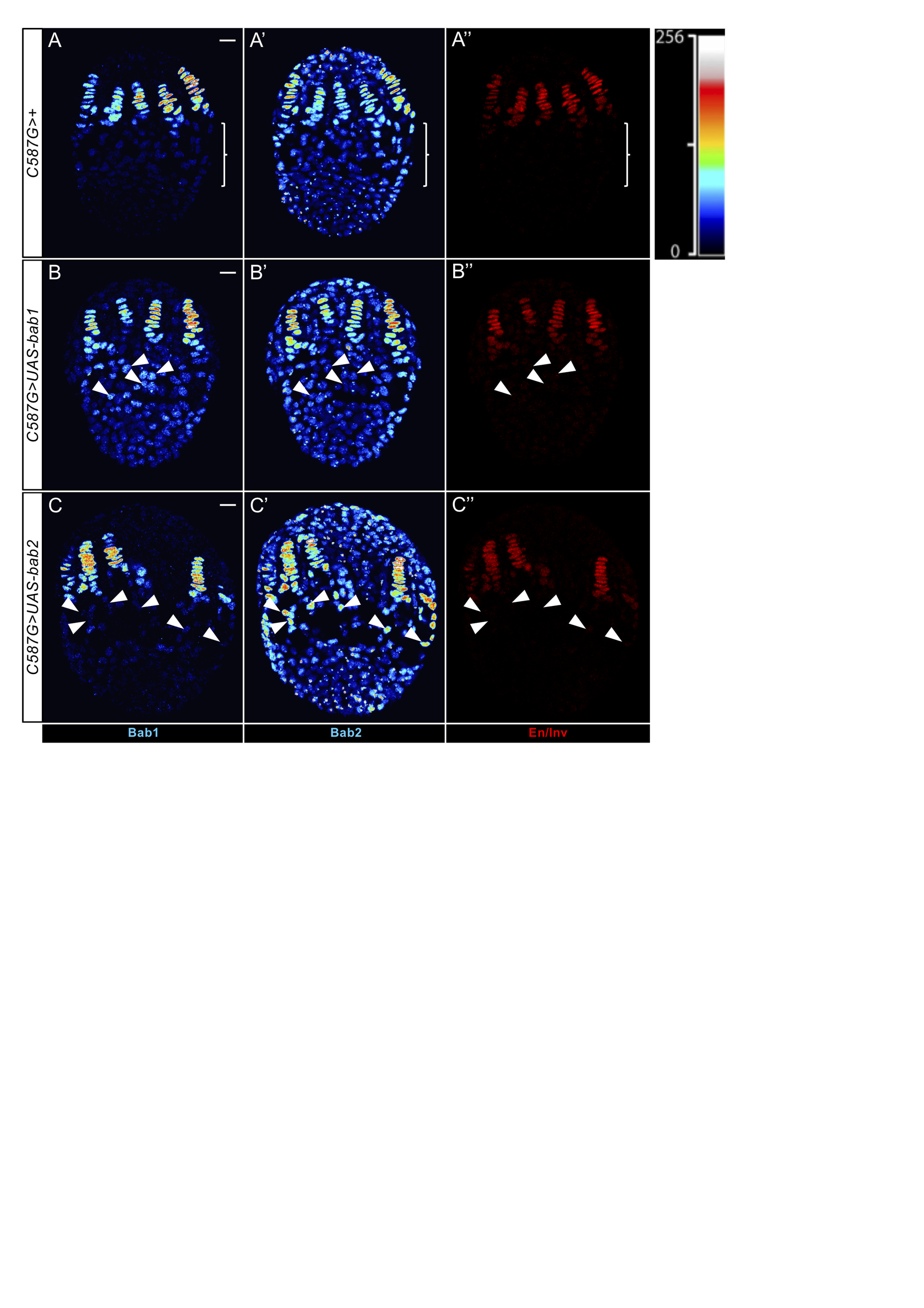

Supplement: S9 Fig — (A-C) Prepupal ovaries from females raised at 25°C, immunostained for Bab1 and Bab2 (Fiji Royal Lookup Table (LUT) indicating signal intensity, inset to the right) and Engrailed/Invected (En/Inv, red). Anterior is up. Scale bars: 10μm. (A) Control ovary (C587G>+) showing the accumulation of Bab1 (high in niche cells and low in Intermingled Cells (ICs, white brackets)). (B) The C587G driver coupled with UAS-bab1 allows increased accumulation of Bab1 in some ICs (arrowheads). (C') The C587G driver coupled with UAS-bab2 also leads to increased accumulation of Bab2 in some ICs (arrowheads). (B',C vs. A',A) No evidence of cross-regulation between Bab1 and Bab2 is observed. (B”,C”) Neither of these transgenes causes the presence of ectopic En/Inv in ICs with an excess of Bab1 or Bab2 (arrowheads) compared to the control (A"). The overall organization of these ovaries does not seem disturbed when compared to the control. (TIF) [file pgen.1009128.s009.tif]

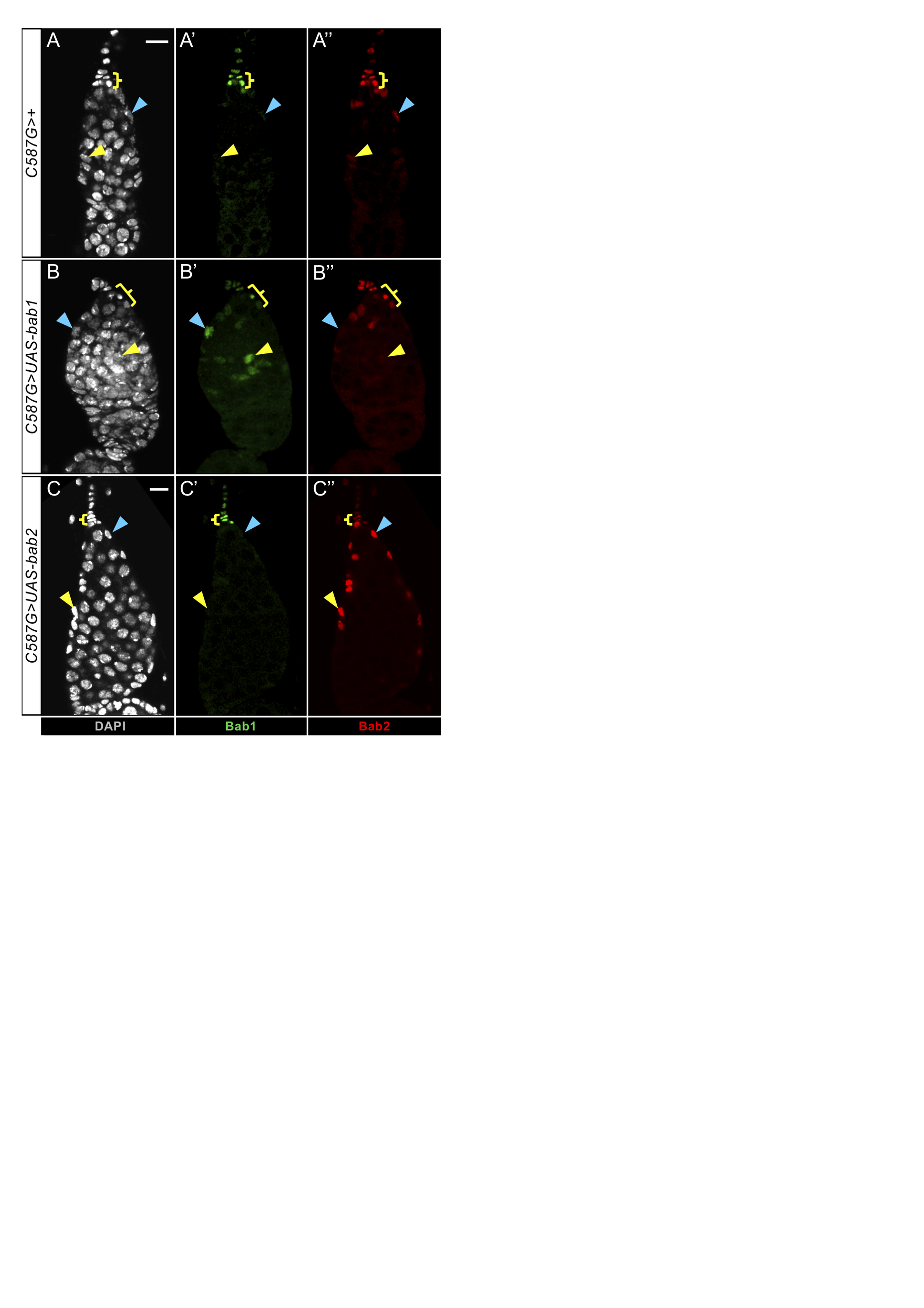

Supplement: S10 Fig — (A-C) Germaria from ovaries of 10-day old females immunostained for Bab1 (green) and Bab2 (red). DAPI nuclear labeling is in grey. Anterior is up. Scale bars: 10μm. (A-A") In the control (C587G>+), Bab1 (A’) and Bab2 (A”) are present in niche cells, mainly in the Cap Cells (CCs) (yellow bracket) and overlying Terminal Filaments (TFs). Bab2 can also be detected faintly in some Escort Cells (ECs) (A”, blue arrowhead) and more posterior somatic cells (A", yellow arrowhead). (B-B”) Ectopic expression of bab1 (C587G>UAS-bab1, B’) or overexpression of bab2 (C587G>UAS-bab2, C") causes elevated accumulation of the corresponding proteins in ECs and prefollicle cells (blue and yellow arrowheads, respectively). Cross regulation between bab1 and bab2 does not occur (B” and C’, blue and yellow arrowheads). (TIF) [file pgen.1009128.s010.tif]
