# Supplementary material for: The Bric-à-Brac BTB/POZ transcription factors are necessary in niche cells for germline stem cells establishment and homeostasis through control of BMP/DPP signaling in the Drosophila melanogaster ovary
Source: PLoS Genet. 2020 Nov 5;16(11):e1009128. doi: 10.1371/journal.pgen.1009128 (PMC7643948; doi:10.1371/journal.pgen.1009128)
Supplement: S1 Text — (DOCX) [file pgen.1009128.s012.docx]

S1 Text**. Supplemental materials and experimental procedures**

**Fly stocks**

We used two short hairpin/miRNA (shmiR) transgenic lines that can specifically target sequences unique to *bab1* and to *bab2* for RNA-interference (RNAi). These “chained” shmiR transgenes are under the cis-regulatory control of Upstream Activating Sequences (UAS). We obtained similar results using lines Id #3-12 and #3-16 [49]

**Experimental conditions**

Developmental staging of larvae: flies were raised under uncrowded conditions. Parents were transferred into a fresh vial to lay eggs for 12 h at 25°C, and were then removed. Vials were then left at 29°C for 48-72h for L2 and early L3 (EL3) and 72-96h to obtain mid third instar (ML3). Late L3 (LL3) stage corresponds to wandering L3 larvae. L2 and EL3 stages were distinguished according to anterior spiracle shape.

For pupal and adult knockdown of *bab2* in *hhG>UAS-bab2^IR^,* or *bab^A128^, G80^TS^, hhG>UAS-bab2^IR^* flies or in *G80^TS^; hhG* control flies, individuals were raised at 18°C from egg laying to 1-day old pupal stage, then shifted at 29°C until adult eclosion and transferred at 31°C for 7 days.

**Immunostaining**

E-Cadherin was detected using the DCAD2 rat antibody (DSHB, 1:100)
